# G3BP1 methylation by PRMT5 facilitates its recruitment of TBK1 for IRF3 activation during host response to foreign ribonucleic acids

**DOI:** 10.3389/fimmu.2026.1888266

**Published:** 2026-07-08

**Authors:** Wen Song, Susana Soo-Yeon Kim, Candice Yam, Joey Teo, Jyue Yuan Lim, Xuezhi Bi, Hong-Hwa Lim, Kong-Peng Lam

**Affiliations:** 1 Singapore Immunology Network (SIgN), Agency for Science, Technology and Research (ASTAR), Singapore, Singapore; 2 Bioprocessing Technology Institute (BTI), Agency for Science, Technology & Research (ASTAR), Singapore, Singapore; 3 Department of Microbiology & Immunology, Yong Loo Lin School of Medicine, National University of Singapore, Singapore, Singapore; 4 School of Biological Sciences, Nanyang Technological University, Singapore, Singapore

**Keywords:** adaptor protein, G3BP1, innate immunity, interferon, RNA-sensing

## Abstract

**Background and aim:**

Ras GTPase-activating protein SH3 domain-binding protein 1 (G3BP1) has been shown to bind cytosolic nucleic acid sensors including retinoic acid-inducible gene I (RIG-I) and cyclic GMP-AMP synthase (cGAS) in innate immunity. As a scaffolding protein enhancing protein-protein and protein-RNA interactions during cellular stress response, we examine whether G3BP1 could bind additional innate immune signaling molecules and investigate the relevance and mechanisms underlying these interactions.

**Methods:**

To define G3BP1 function in dsRNA-triggered anti-viral signaling and identify additional interacting molecules, we generated siRNA- and CRISPER-Cas9-mediated G3BP1-knockdown and knockout HEK293T cells, conducted biochemical experiments in human and murine cells including co-immunoprecipitation, domain mutagenesis and mapping, as well as LC-MS/MS analyses to study protein post-translational modifications. We also performed p(I:C)-stimulation assays to measure interferon production, pharmacological inhibition studies to determine enzyme-substrate specificity, as well as confocal immunofluorescence microscopy to examine protein subcellular localization.

**Results:**

Our study reveals a role for G3BP1 in binding TBK1 and IRF3, two important molecules in innate immune signaling leading to the production of IFN-β. Deficiency in G3BP1 reduces TBK1–IRF3 complex formation and affects IRF3 phosphorylation, nuclear translocation, and the production of IFN-β during p(I:C) stimulation while the phosphorylation of TBK1 was largely preserved. We further show that G3BP1 constitutively associates with IRF3 and recruits TBK1 upon p(I:C) stimulation. These findings suggest that G3BP1 acts as a scaffold to recruit activated TBK1 and facilitates its activation of IRF3. Domain mapping experiments indicated that the C-terminal RGG region of G3BP1 is critical for binding IRF3 and TBK1. LC–MS/MS analysis revealed human G3BP1 to be arginine-methylated at R435 and R460 residues upon p(I:C)-stimulation, and mutagenesis experiments indicated that R460 is required for its efficient binding of TBK1. We further show that the arginine methyltransferase PRMT5 associates with and promotes the symmetric arginine-dimethylation of G3BP1 and pharmacological inhibition of PRMT5 impaired G3BP1–TBK1 interaction.

**Conclusions:**

Together, our data indicate that G3BP1 could bind PRMT5, TBK1 and IRF3 and reveal G3BP1 as a signaling hub that constitutively binds IRF3 and is arginine-methylated by PRMT5 to recruit TBK1 for IRF3 phosphorylation and activation and the induction of interferon production during host antiviral response.

## 1 Introduction

The Ras GTPase-activating SH3-domain-binding protein 1 (G3BP1) is an evolutionarily conserved, multidomain RNA-binding protein best known as a core component of stress granules (SGs), which are cytoplasmic ribonucleoprotein assemblies that form in response to cellular stress, including heat shock and viral infection (1, 2). During infection, a specialized subset of SGs, termed antiviral stress granules (avSGs), can sequester viral RNA and thereby restrict viral replication (3, 4). G3BP1 is essential for avSG assembly, and accordingly, many viruses have evolved strategies to antagonize G3BP1 function, including the use of viral proteases or non-coding RNAs to disrupt granule formation and host antiviral defense (5). These observations place G3BP1 at a critical interface between cellular stress adaptation and antiviral protection.

In parallel with activating stress responses, viral RNA can also trigger innate immune signaling that culminates in the production of type I interferon. Cytosolic viral double-stranded RNA is recognized by retinoic acid-inducible gene I (RIG-I), which signals through the adaptor MAVS to recruit the kinase TBK1, leading to the phosphorylation and activation of the transcription factor IRF3 (6, 7). The association of TBK1 with IRF3 is required for the phosphorylation of IRF3, which activates its dimerization, nuclear translocation and the subsequent induction of IFN-β expression (8–10). Given that stress responses and innate immune signaling are activated concurrently during viral infection, it is plausible that key components of SGs may also participate more directly in anti-viral signal transduction.

Consistent with this idea, accumulating evidence suggests that G3BP1 could function beyond its structural role in nucleating SG formation and directly participates in innate immune signaling. G3BP1 has been shown to act as a co-receptor for RIG-I in the recognition of viral dsRNA (6), to promote cGAS association with viral DNA to activate DNA-triggered type I interferon signaling (11, 12), and to support the efficient translation of interferon-stimulated gene mRNAs (13). In addition, genomic and siRNA-based screening studies had further implicated G3BP1 in the optimal production of IFN-β (14). Together, these findings suggest that G3BP1 could have a broader role in the regulation of antiviral immunity. However, despite these findings, whether G3BP1 could directly serve as a signaling scaffold to bind additional innate immune signaling molecules is unknown. In particular, whether G3BP1 could promote the assembly and activation of the critical downstream TBK1–IRF3 complex remains unclear.

Structurally, G3BP1 contains an N-terminal nuclear transport factor 2 (NTF2)-like domain, an acidic region, an RNA-recognition motif (RRM) and a C-terminal arginine-glycine-rich (RGG) region. Arginine-rich RGG motifs are common targets of post-translational methylation by protein arginine methyltransferases (PRMTs), a modification known to regulate protein interactions, localization and stress granule dynamics (5, 15–18). The presence of this RGG motif raises the possibility that arginine methylation of G3BP1 may also regulate its innate immune signaling function. However, whether this is indeed the case, is not demonstrated.

Here, we show that G3BP1 acts as an adaptor to bind both TBK1 and IRF3. It associates with IRF3 constitutively and binds TBK1 upon innate stimulation, leading to the subsequent phosphorylation and activation of IRF3 for IFN-β production. We identify the C-terminal RGG region of G3BP1 as essential for its interaction with IRF3 and TBK1. Furthermore, we demonstrate that the R460 residue within G3BP1 RGG domain, and arginine methylation mediated preferentially by PRMT5, are required for efficient G3BP1 binding of TBK1 and the initiation of downstream innate immune signaling for IFN-β production.

## 2 Materials and Methods

### 2.1 Cells and generation of G3BP1-knockout cells

HEK293T and RAW264.7 cells were maintained in DMEM and RPMI 1640 medium, respectively, supplemented with 10% FBS and penicillin–streptomycin, in a humidified incubator containing 5% CO_2_. RAW264.7 cells were used for the initial mouse G3BP1 LC–MS/MS analysis and for additional validation of G3BP1–IRF3/TBK1 interactions and IFN-β reporter activity in a murine cell background, as indicated in the corresponding Results and Supplementary Figures. G3BP1-KO HEK293T cells were generated as previously described (19) by transfection of pSB vectors encoding the G3BP1-targeting guide RNAs 5′-TACCACACCATCATTTAGCG-3′ and 5′-GAATCATCAGGTACCACCTC-3′ together with transposase using Lipofectamine 3000, followed by puromycin selection for 5 d (20).

### 2.2 Antibodies and reagents

For western blotting, the following primary antibodies were used: anti-G3BP1 (Bethyl Laboratories, A302-033A), anti-TBK1/NAK (D1B4, Cell Signaling Technology, #3504), anti-IRF3 (D83B9, Cell Signaling Technology, #4302), anti-phospho-TBK1/NAK (Ser172) (D52C2, Cell Signaling Technology, #5483S), anti-phospho-IRF3 (Ser396) (D6O1M, Cell Signaling Technology, #29047), anti-GAPDH (14C10, Cell Signaling Technology, #2118), anti-PRMT1 (A33, Cell Signaling Technology, #2449S), anti-PRMT5 (D5P2T, Cell Signaling Technology, #79998), anti-asymmetric dimethyl arginine motif [ADME-R] MultiMab rabbit monoclonal antibody mix (Cell Signaling Technology, #13522), symmetric di-methyl arginine recombinant rabbit monoclonal antibody (9E6M4, Thermo Fisher Scientific, MA5-55511), anti-HA (Sigma-Aldrich, H6908) and ANTI-FLAG M2-Peroxidase (HRP) antibody (Sigma-Aldrich, A8592). For immunoblotting of whole-cell lysates, anti-rabbit IgG, HRP-linked antibody (Cell Signaling Technology, #7074) was used as the secondary antibody. For immunoblotting of immunoprecipitated samples, mouse anti-rabbit IgG (Conformation Specific) (L27A9) monoclonal antibody (HRP conjugate) (Cell Signaling Technology, #5127) was used where indicated to minimize interference from denatured IgG heavy chain and light chain.

For immunofluorescence staining, anti-IRF3 (D9J5Q) mouse monoclonal antibody (Cell Signaling Technology, #10949) was used as the primary antibody, and Alexa Fluor 488-conjugated goat anti-mouse IgG (H+L) was used as the secondary antibody. Cell nuclei were counterstained with DAPI (Sigma-Aldrich, #D9542). Cell Lysis Buffer (10×) (Cell Signaling Technology, #9803), Protease/Phosphatase Inhibitor Cocktail (Cell Signaling Technology, #5872), bovine serum albumin (BSA) (Capricorn Scientific, BSA-1S), non-fat dry milk (Cell Signaling Technology, #9999), WesternBright Sirius Chemiluminescent Detection Kit (Advansta, K-12043-D10) and ANTI-FLAG M2 agarose affinity gel (Sigma-Aldrich) were used as indicated. The PRMT inhibitor EPZ015666 (MedChemExpress, HY-100235) and AMI-1 (Santa Cruz Biotechnology, CAS 20324-87-2) were dissolved in sterile ultrapure water.

### 2.3 Plasmids construction, mutagenesis and transfection

Human or mouse cDNAs encoding G3BP1 transcript variant X1, TBK1 transcript variant X1 and IRF3 transcript variant 1 were amplified by PCR from HEK293T cDNA and subcloned into pxj40-HA or pxj40-FLAG using HindIII and KpnI to generate in-frame tagged expression constructs. G3BP1 truncation mutants corresponding to fragments A–F were generated by PCR using specific primers, labeling as G^A^, G^B^, G^C^, G^D^, G^E^, G^F^. The R435K and R460K point mutants of G^F^ were generated by site-directed mutagenesis using mutation-specific primers, labeling as G^F_R435K^, G^F_R460K^. TBK1 truncation mutants corresponding to fragments A–C were generated by PCR using specific primers, labeling as T^A^, T^B^, T^C^. All the sequences mentioned are provided in *Supplementary Materials*. HEK293T cells were transfected with recombinant plasmids or mutant constructs using Lipofectamine 3000 (Thermo Fisher Scientific).

### 2.4 si-RNAs and transfection

Control siRNA and siRNAs targeting G3BP1 were purchased from Santa Cruz Biotechnology (sc-75076). For siRNA transfection, 1 × 10^6^ cells were seeded per well in 6-well plates and transfected with Lipofectamine RNAiMAX Transfection Reagent (Thermo Fisher Scientific, #13778030). After 48 h, cells were transfected with p(I:C) (InvivoGen) using Lipofectamine 3000 (Thermo Fisher Scientific) for 9 h, as indicated.

### 2.5 Immunoprecipitations and immunoblotting

Cells were transfected with plasmids and treated with or without p(I:C), and whole cell lysates were used for detecting endogenous protein or protein-protein interactions. Cells were lysed in 1× cell lysis buffer containing protease/phosphatase inhibitor (1:100), sonicated, and clarified by centrifugation. Whole-cell lysates (50 μg) were analyzed by SDS–PAGE and immunoblotting using standard procedures. For immunoprecipitation, 1 mg of lysate was incubated with the indicated antibody overnight at 4 °C and captured with protein A/G Plus-agarose (Santa Cruz Biotechnology, sc-2003). FLAG-tagged proteins were immunoprecipitated using ANTI-FLAG M2 Affinity Gel. Immunoreactive bands were visualized by chemiluminescence. A conformation-specific anti-rabbit HRP-conjugated secondary antibody was used for immunoblots of immunoprecipitates where indicated to reduce detection of IgG heavy chain.

### 2.6 IFN-β luciferase reporter assay

WT and G3BP1-KO HEK293T cells were seeded in 12-well plates at a density of 1 × 10^5^ cells per well and co-transfected with empty vector or expression constructs encoding Flag/HA-IRF3, Flag/HA-TBK1, HA-G^F^, HA-G^F_R435K^ or HA-G^F_R460K^, together with the IFN-β luciferase reporter plasmid (900 ng per well) and a Renilla internal control plasmid (100 ng per well; Promega), using Lipofectamine 3000. The *IFNB1* promoter reporter was kindly provided by D. Wang (Zhejiang University, Zhejiang, China). Firefly and Renilla luciferase activities were measured 48 h after transfection using a dual-luciferase reporter assay system (Promega), as previously described (21).

### 2.7 Immunofluorescence staining and confocal microscopy

WT and G3BP1-KO HEK293T cells were left untreated or stimulated with p(I:C), as indicated. Cells were fixed with 4% paraformaldehyde for 20 min, washed with PBS, permeabilized with 0.5% Triton X-100 for 10 min, and blocked with 5% BSA for 1 h. Cells were then incubated overnight at 4 °C with anti-IRF3 antibody (D9J5Q), followed by Alexa Fluor 488-conjugated goat anti-mouse IgG (H+L). Nuclei were counterstained with DAPI. After washing with PBS, cells were mounted with ProLong Gold antifade reagent and imaged using an Olympus FV3000 confocal microscope under 40× or 60× objectives.

### 2.8 EPZ015666 and AMI-1 treatment

Cells were treated at approximately 50–60% confluency with EPZ015666 (50 μM) or AMI-1 (100 μM) for 48 h. Control cells received an equivalent volume of DMSO as vehicle. For stimulation, p(I:C) was added 8 h before cell collection. Cells were then harvested for immunoblotting and immunoprecipitation analyses.

### 2.9 Cell-based methylation analysis

HEK293T cells were co-transfected with G3BP1 and PRMT1 or PRMT5 expression constructs. Whole-cell lysates were subjected to immunoblot analysis to assess changes in cellular arginine methylation using antibodies against asymmetric or symmetric dimethylarginine. Expression of G3BP1, PRMT1, PRMT5, SDMA, ADMA and other indicated proteins was confirmed by immunoblotting.

### 2.10 Quantitative RT-PCR

Total RNA from WT and G3BP1-KO HEK293T cells was extracted using the RNeasy Mini Kit (Qiagen), and cDNA was synthesized using the RevertAid First Strand cDNA Synthesis Kit (Thermo Scientific, K1622). Quantitative PCR was performed on an Applied Biosystems 7500 real-time PCR system. The following genes were analyzed: *IFNB1* (IFN-β) and *GAPDH*. Primer sequences are listed in *Supplementary Materials*. Relative expression levels were determined by normalizing target gene expression to *GAPDH*.

### 2.11 Mass spectrometry and data analysis

For the analysis of mouse and human G3BP1 by LC–MS/MS analysis, FLAG-tagged G3BP1 was immunoprecipitated using anti-FLAG M2 agarose affinity gel (Sigma-Aldrich), resolved by SDS–PAGE and visualized by Coomassie staining. Gel bands corresponding to G3BP1 were excised, digested in-gel with chymotrypsin or pepsin, and analyzed via LC–MS/MS as previously described (22) using an LTQ-Orbitrap Elite (mouse G3BP1) Orbitrap Eclipse Tribrid (Human G3BP1) mass spectrometer coupled to ACQUITY UPLC M class system.

MS data were analyzed with PEAKS Studio 13. Human G3BP1 LC–MS/MS data were searched against the human Swiss-Prot database, whereas the original mouse G3BP1 LC–MS/MS data were searched against the Mus musculus Swiss-Prot database. Peptide and fragment ion mass tolerances were set to ±5 ppm and ±0.02 Da, respectively. Carbamidomethylation of cysteine was included as a fixed modification, oxidation of methionine, phosphorylation of serine, threonine and tyrosine and di-methylation or mono-methylation of lysine/arginine, as well as tri-methylation or acetylation of lysine were selected as dynamic and variable modifications. One missed cleavage was allowed for both chymotrypsin and pepsin digestions. Percentage of modified peptide area (total area of the features associated with the corresponding di-methylated peptide) over G3BP1 protein area (total area of all peptide features from unique supporting peptides in the protein) were calculated. Three independent LC-MSMS runs from chymotrypsin or pepsin digested peptides were performed.

### 2.12 Statistical analysis

Quantitative data are represented as mean ± SD and p values of less than 0.05 were considered statistically significant. Unpaired two-tailed student’s t test were calculated using prism software (Prism; GraphPad Software, San Diego, CA). Confocal images were processed using Fiji/ImageJ (23). For presentation purposes, linear adjustments of brightness and contrast were applied equally across all images within the same experiment. Channel merging was performed in Fiji/ImageJ.

## 3 Results

### 3.1 G3BP1 positively regulates p(I:C)-induced IFN-β production

**Figure 1:**
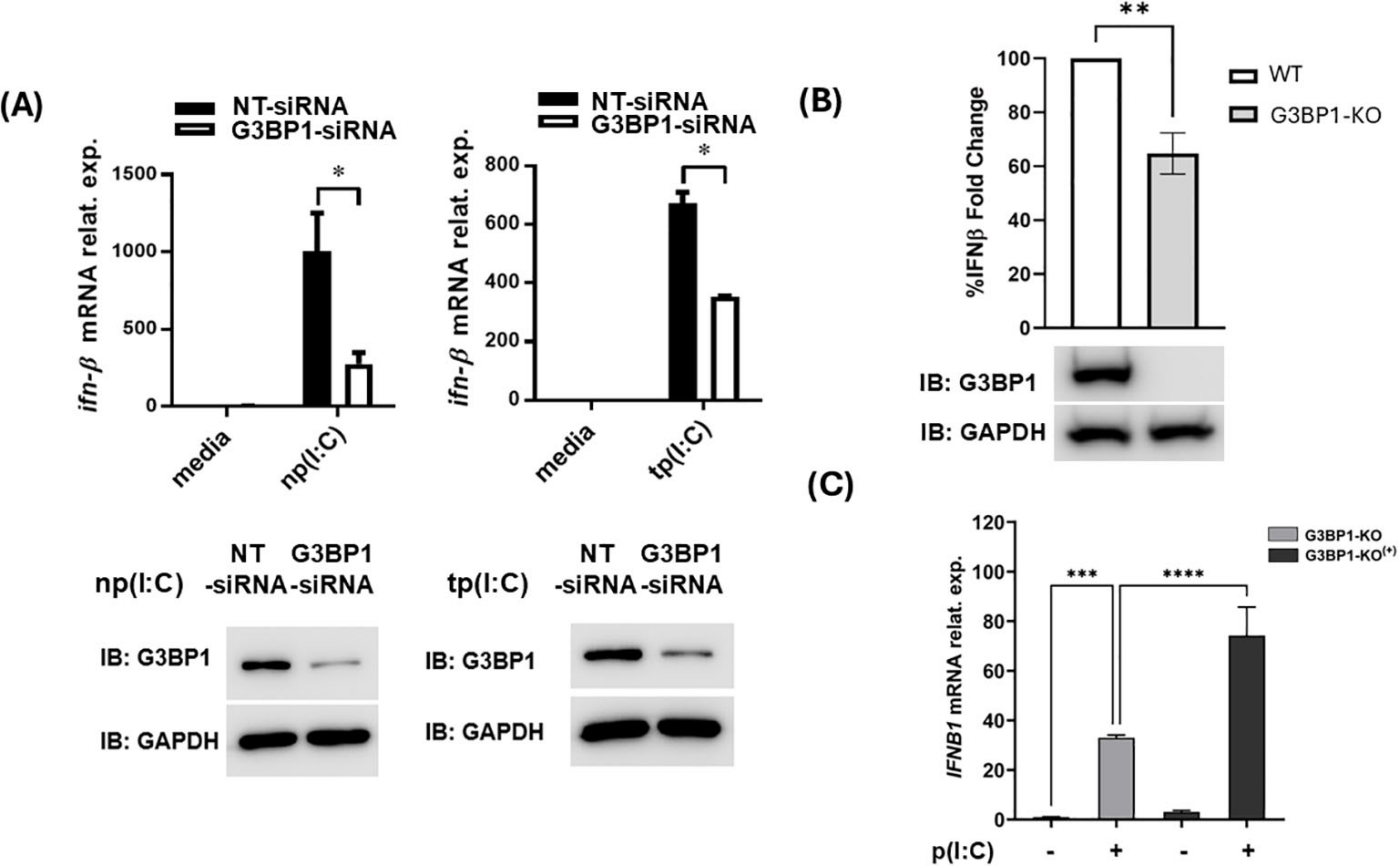
G3BP1 is required for optimal p(I:C)-induced *IFNB1* Mrna production in HEK293T cells. **(A)**, G3BP1 knockdown via siRNA in HEK293T cells reduced *IFNB1* mRNA induction in HEK293T cells stimulated with naked p(I:C) [np(I:C)] or transfected p(I:C) [tp(I:C)], as determined by qRT-PCR. G3BP1 knockdown efficiency was verified by immunoblotting, with GAPDH as the loading control. **(B)**, G3BP1-KO HEK293T cells exhibited reduced *IFNB1* mRNA induction compared with control cells after 9 h of p(I:C) stimulation. G3BP1 deficiency was verified by immunoblotting, with GAPDH as the loading control. **(C)**, Reintroduction of G3BP1 restored p(I:C)-induced *IFNB1* mRNA expression in G3BP1-KO HEK293T cells, as determined by qRT-PCR. *p ≤ 0.05; **p ≤ 0.01;***p ≤ 0.001;****p ≤ 0.0001; ns, not significant.

G3BP1 nucleates stress granule formation (19) and in multiple viral infection models, has been shown to sequester and restrict viral RNA translation and viral replication in host cells (24–26). We previously showed that G3BP1 could bind and act as a co-sensor of RIG-I (6). To examine whether G3BP1 plays a role in IFN-β induction, we first attenuated G3BP1 expression in HEK293T cells using small interfering RNA (siRNA) (**Figure 1A**). We found that a reduction in G3BP1 expression led to diminished *IFNB1* mRNA induction in HEK293T cells following their stimulation with either naked polyinosinic:polycytidylic acid (np(I:C)) or transfected p(I:C) that was stabilized with poly-L-lysine in carboxymethylcellulose (tp(I:C); **Figure 1A**). Because np(I:C) and tp(I:C) preferentially activate the TLR3 and RLR pathways, respectively (27), these data suggested that G3BP1 may function at a signaling step common to both pathways during IFN-β induction. Hence, G3BP1 is unlikely to act exclusively downstream of a single RNA sensor, but may contribute to a signaling step common to multiple nucleic-acid pathways such as the one converging on TBK1–IRF3 activation.

To simplify experimental designs, we used tp(I:C) in subsequent experiments and annotated it as p(I:C). We also generated G3BP1-knockout (G3BP1-KO) HEK293T cells using CRISPR/Cas9 approach and analyzed its *IFNB1* mRNA expression before and after p(I:C) stimulation. HEK293T cells mounted a robust innate immune response (28), whereas G3BP1-KO cells showed markedly reduced *IFNB1* mRNA induction at 9 h after stimulation (**Figure 1B**). Re-expression of *G3BP1* in G3BP1-KO HEK293Tcells restored *IFNB1* mRNA expression levels during p(I:C) stimulation (**Figure 1C**). These findings indicated that G3BP1 positively regulates p(I:C)-triggered IFN-β production.

### 3.2 G3BP1 constitutively binds IRF3 and associates with TBK1 upon p(I:C)-stimulation

**Figure 2:**
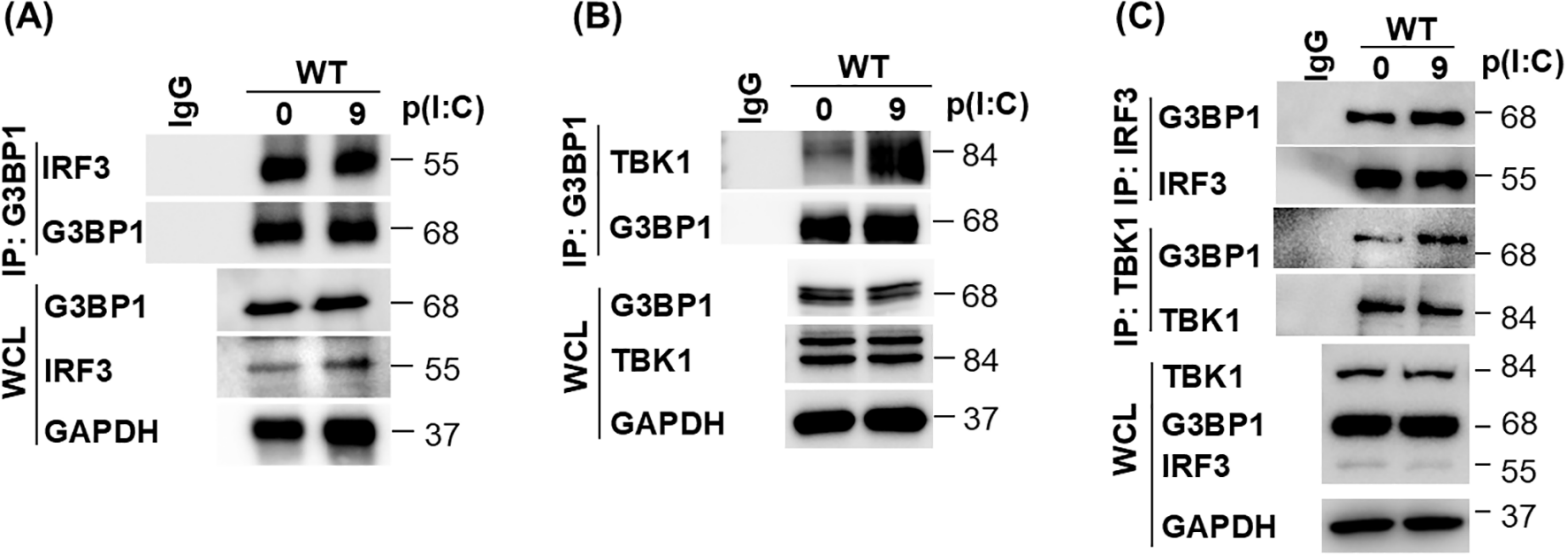
G3BP1 binds IRF3 constitutively and TBK1 upon p(I:C) stimulation. HEK293T cells were left untreated or stimulated with p(I:C), and whole-cell lysates (WCL) were subjected to co-immunoprecipitation (Co-IP) and immunoblot analysis. Data shown are representative of more than 3 independent experiments. **(A)**, G3BP1 immunoprecipitation followed by immunoblotting for IRF3. **(B)**, G3BP1 immunoprecipitation followed by immunoblotting for TBK1. **(C)**, Reciprocal Co-IP using anti-IRF3 or anti-TBK1 antibodies, followed by immunoblotting for G3BP1, IRF3 and TBK1. Input WCL were analyzed for G3BP1, TBK1, IRF3 and GAPDH as indicated. Experiment shown are representative of over 5 independent analyses.

We previously showed that G3BP1 could bind RIG-I (6). Since G3BP1 is a scaffolding protein possessing multiple motifs known for protein-protein interactions, we explored if G3BP1 could bind other signaling molecules downstream of RIG-I signaling such as the TBK1 kinase and IRF3 transcription factor. We performed endogenous co-immunoprecipitation (Co-IP) analyses of HEK293T cells with or without p(I:C) stimulation and found readily detectable association of IRF3 with G3BP1 under basal conditions, and this interaction was maintained after stimulation (**Figure 2A**). By contrast, association of TBK1 with G3BP1 was weak in unstimulated cells but was markedly enhanced following p(I:C) treatment (**Figure 2B**). These results suggest that G3BP1 constitutively associates with IRF3, whereas its interaction with TBK1 is induced in a stimulus-dependent manner.

To confirm these interaction patterns, we performed reciprocal co-immunoprecipitations using antibodies against IRF3 or TBK1 (**Figure 2C**). Consistent with the G3BP1 pull-down experiments, G3BP1 was detected in IRF3 immunoprecipitates both before and after stimulation. In contrast, the association of G3BP1 with TBK1 was low under basal conditions and was increased following p(I:C) stimulation. To examine whether a similar interaction pattern is observed in a murine immune-cell background, we performed parallel co-immunoprecipitation analyses in RAW264.7 cells. Consistent with the HEK293T results, G3BP1 associated with IRF3 and showed enhanced association with TBK1 following p(I:C) stimulation in RAW264.7 cells (**Supplementary Figure 1**). Together, these data support a model in which IRF3 constitutively associates with G3BP1, whereas TBK1 is recruited to G3BP1 in a stimulus-dependent manner.

### 3.3 G3BP1 is required for efficient TBK1–IRF3 coupling and IRF3 activation

TBK1 is known to be phosphorylated during antiviral signaling, and it in turn phosphorylates IRF3 for nuclear translocation to activate IFN-β production (8, 21). The interaction patterns described above (**Figure 2**) raised the possibility that G3BP1 could facilitate the productive coupling between TBK1 and IRF3. We therefore proceed to examine if loss of G3BP1 affects the activation of TBK1 and IRF3 and the formation of TBK1–IRF3 complex necessary for IRF3 activation.

**Figure 3:**
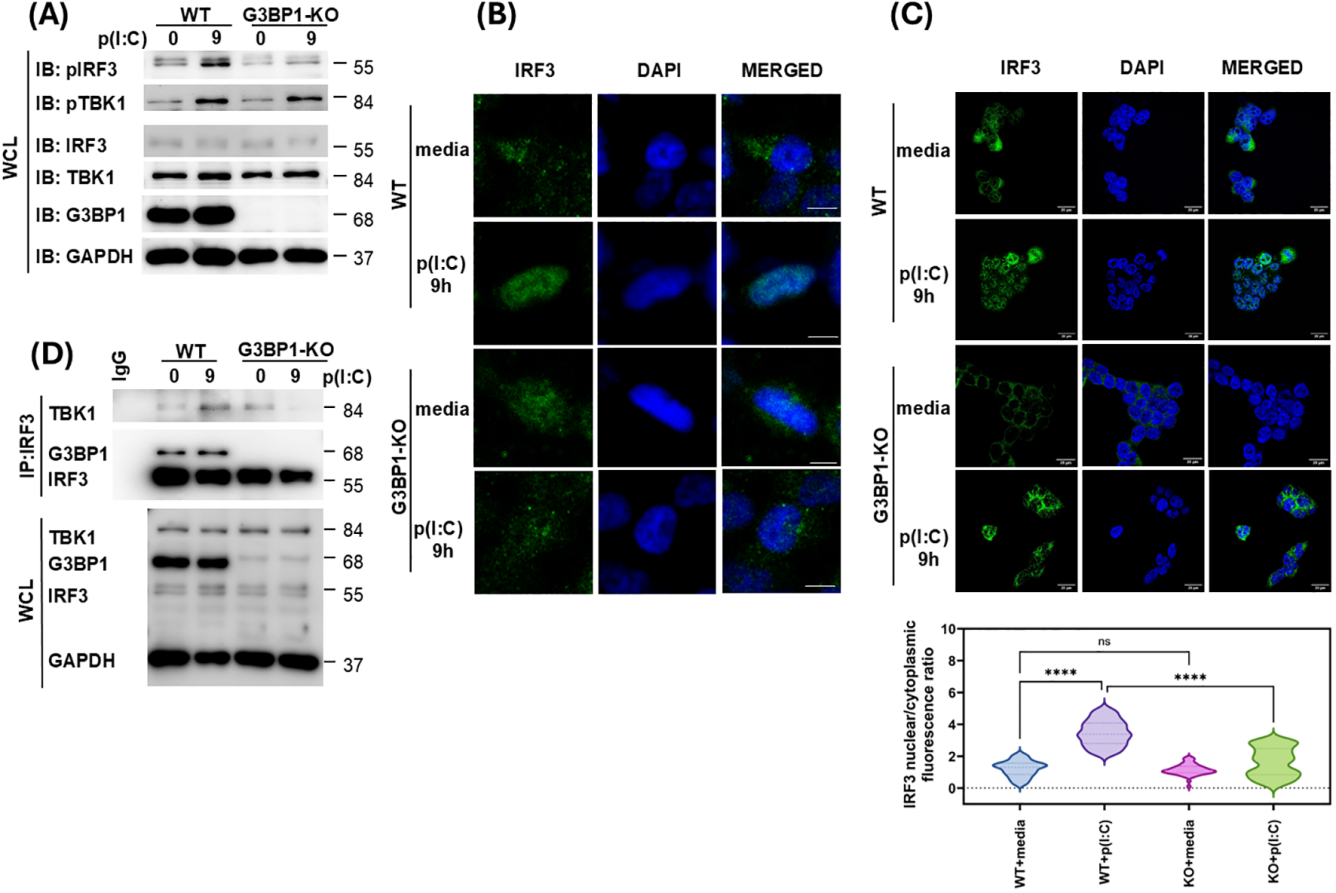
G3BP1 deficiency impairs IRF3 activation and nuclear translocation and affects TBK1/IRF3 complex formation. **(A)**, Immunoblot analyses of IRF3 and TBK1 activation in HEK293T wildtype (WT) and G3BP1-knockout (KO) cells that were untreated or stimulated with p(I:C). Whole-cell lysates (WCL) were subjected to immunoblotting with antibodies against phospho (p)-IRF3, pTBK1, IRF3, TBK1, G3BP1 and GAPDH (as loading control). Data shown are representative of more than 3 independent experiments. **(B)**, Confocal microscopy of IRF3 nuclear localization in untreated or p(I:C)-stimulated WT and G3BP1-KO HEK293T cells at 9 h post-treatment. Nuclei were stained with DAPI. Scale bar, 10 μm. **(C)**, Confocal images and quantification of IRF3 nuclear translocation in multiple WT and G3BP1-KO HEK293T cells. Nuclear-to-cytoplasmic IRF3 fluorescence intensity ratios were quantified under the indicated conditions. Scale bars, 20 mm. ns, not significant; ****, p<0.0001. **(D)**, Co-IP analysis of endogenous TBK1–IRF3 interaction in p(I:C)-stimulated WT and G3BP1-KO HEK293T cells, examined by IRF3 Co-IP followed by immunoblotting for TBK1 and G3BP1. Input lysates were analyzed for G3BP1, TBK1, IRF3 and GAPDH.

Immunoblot analysis showed that p(I:C) stimulation induced robust IRF3 phosphorylation in wild-type (WT) HEK293T cells and this response was markedly attenuated in G3BP1-KO cells (**Figure 3A**). By contrast, p(I:C)-induced TBK1 phosphorylation remained equivalent in G3BP1-sufficient and-deficient cells, suggesting that G3BP1 is dispensable for TBK1 phosphorylation per se but is required for efficient activation of IRF3.

Because phosphorylation of IRF3 by activated TBK1 promotes its nuclear translocation (8), we also assessed the subcellular localization of IRF3 by confocal microscopy, as shown in **Figures 3B, C**. In WT cells, p(I:C) stimulation induced clear nuclear accumulation of IRF3, whereas this subcellular redistribution was substantially impaired in G3BP1-KO cells. Quantification of nuclear-to-cytoplasmic IRF3 fluorescence intensity ratios confirmed that p(I:C)-induced IRF3 nuclear translocation was significantly reduced in G3BP1-KO cells (**Figure 3C**). Thus, loss of G3BP1 has no major effect on TBK1 phosphorylation but compromises IRF3 phosphorylation and nuclear translocation.

We then asked whether G3BP1 influences the physical coupling between TBK1 and IRF3. Endogenous co-immunoprecipitation of IRF3 revealed readily detectable association of TBK1 with IRF3 in p(I:C)-stimulated WT cells, whereas this interaction was clearly reduced in G3BP1-KO cells (**Figure 3D**). G3BP1 was detected in IRF3 immunoprecipitates in WT cells with or without p(I:C) stimulation. These results indicate that G3BP1 may be required for the assembly of the TBK1–IRF3 signaling complex that promotes IRF3 activation and its nuclear translocation following p(I:C) stimulation.

To complement these biochemical interaction assays with spatial information, we performed time-resolved confocal immunofluorescence analyses of G3BP1 with IRF3 or TBK1 at 0, 1, 3, 6 and 9 h after p(I:C) stimulation (**Supplementary Figures 2**, **3**). IRF3 progressively accumulated in the nucleus following stimulation, whereas G3BP1 remained predominantly cytoplasmic throughout the time course. A cytoplasmic pool of IRF3 remained detectable and showed spatial association with G3BP1 during stimulation, as supported by Pearson’s correlation analysis. These observations suggest that G3BP1 does not co-translocate into the nucleus with IRF3 but remains mainly within the cytoplasmic signaling compartment where it may support IRF3 activation (**Supplementary Figure 2**). By contrast, G3BP1–TBK1 spatial association was weak at baseline but increased after p(I:C) stimulation, consistent with stimulus-induced recruitment of TBK1 to G3BP1. These time-resolved imaging data support a model in which G3BP1 remains predominantly cytoplasmic, where it constitutively associates with IRF3 and promotes stimulus-induced recruitment of TBK1 during RNA-triggered signaling (**Supplementary Figure 3**).

Together, our data in **Figures 2**, **3** suggest that G3BP1 functions as a scaffolding protein, constitutively binding IRF3 and associating with activated TBK1 upon p(I:C) stimulation, to facilitate the formation of the TBK1/IRF3 complex for subsequent IRF3 phosphorylation and nuclear translocation to activate IFN-β production.

### 3.4 The C-terminal RGG region of G3BP1 binds TBK1 and IRF3 and enhances IFN-β production

**Figure 4:**
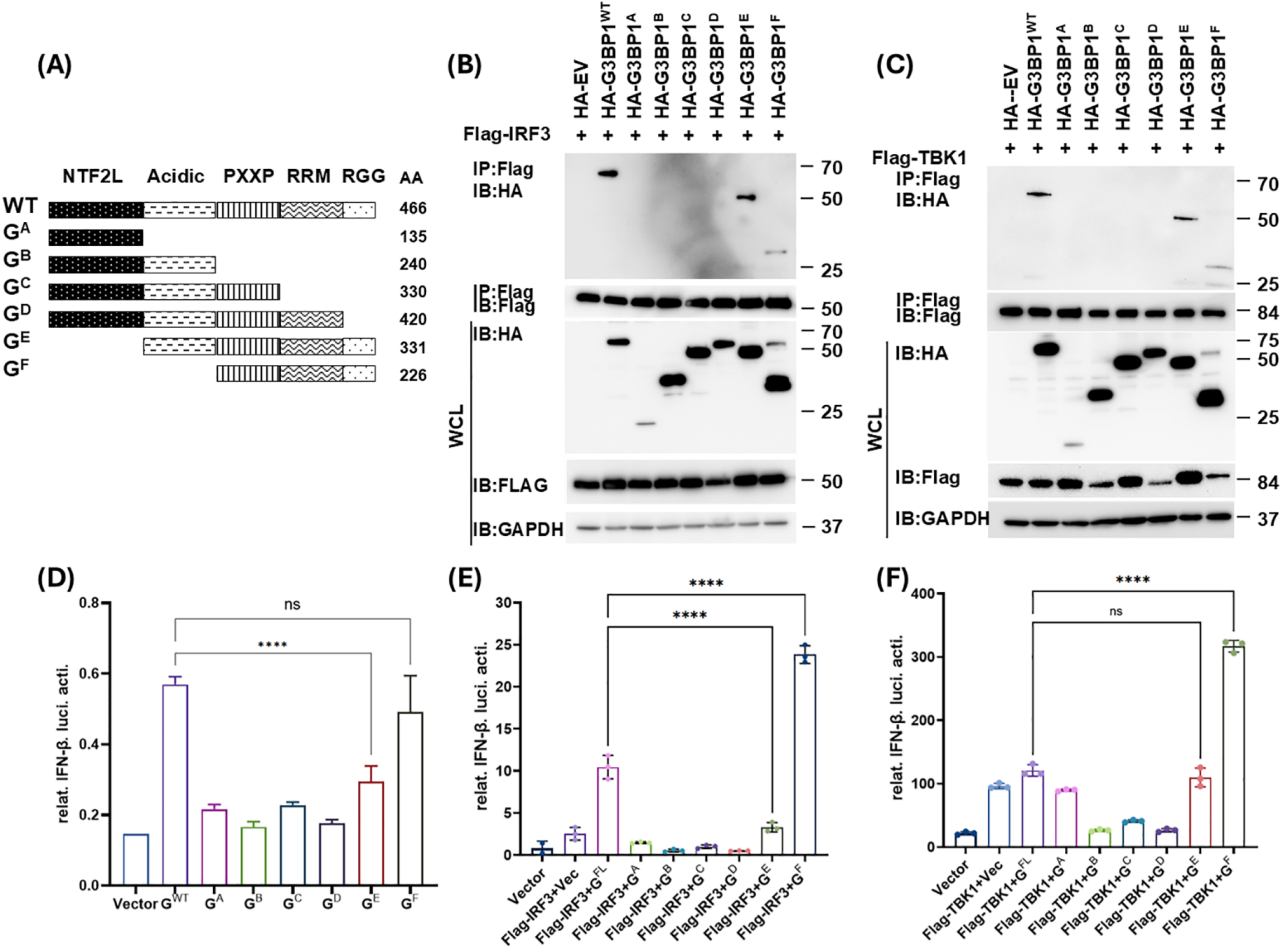
Mapping of G3BP1 domain required for TBK1 and IRF3 association and IFN-β reporter activation. **(A)**, Schematic representation of WT G3BP1, its various domains, and various truncation mutants generated. Numbers beside each construct indicate the number of amino acid (AA) residues. **(B,C)**, Immunoblot analysis of WT and various G3BP1 truncation mutants binding of IRF3 **(B)** or TBK1 **(C)**. HEK293T cells were transfected with the indicated HA- and FLAG-tagged constructs, followed by immunoprecipitation and immunoblotting with the relevant antibodies as indicated. WCL were analyzed to verify expression of the transfected constructs. **(D)**, IFN-β luciferase reporter assay in HEK293T cells transfected with the indicated G3BP1 truncation constructs. **(E, F)**, IFN-β reporter activity in HEK293T cells co-transfected with G3BP1 truncation constructs and Flag-IRF3 **(E)**, or Flag-TBK1 **(F)**. ****p ≤ 0.0001; ns, not significant.

Since G3BP1 could bind both TBK1 and IRF3 (**Figure 2**), we sought to define the region of G3BP1 required for their associations. We generated a series of G3BP1 truncation mutants possessing different combinations of its NTF2-like, acidic, PXXP, RRM and RGG regions (24), as shown in **Figure 4A**. We first examined the interaction of these variants with IRF3 by co-immunoprecipitation of over-expressed proteins in HEK293T cells. Full-length G3BP1 was detected in Flag–IRF3 immunoprecipitates, confirming their association (**Figure 4B**). Among the truncation mutants examined, strong association with IRF3 was seen primarily with G^E^ and G^F^ variants, whereas constructs lacking the C-terminal RGG-containing region showed no detectable association. These data indicate that IRF3 associates with the RGG domain of G3BP1.

We also examined the interaction between G3BP1 truncation variants and IRF3 in G3BP1-KO HEK293T cells and observed similar results (**Supplementary Figure 4**). We further tested these interactions in RAW264.7 cells. Consistent with the HEK293T data, RGG-containing G3BP1 fragments retained IRF3-binding activity in RAW264.7 cells, whereas constructs lacking the C-terminal RGG-containing region showed reduced or undetectable association with IRF3 (**Supplementary Figure 5A**).

We performed similar Co-IP experiments with over-expressed TBK1 and various G3BP1 mutants in HEK293T cells. Full-length G3BP1 Co-IP with TBK1, and as observed with IRF3, only the G^E^ and G^F^ variants could bind TBK1 (**Figure 4C**). Together, the data indicates that G3BP1 binds TBK1 via its C-terminal RGG domain. Similarly, RGG-containing G3BP1 fragments associated with TBK1 in RAW264.7 cells, whereas constructs lacking this region showed reduced TBK1 association (**Supplementary Figure 5B**). These murine and human cell data support the conserved requirement of the G3BP1 C-terminal RGG region for interaction with IRF3 and TBK1.

To determine whether the RGG-domain-dependent interactions translated into functional enhancement of interferon signaling, we examined the effect of G3BP1 truncation variants on IFN-β reporter activity. Using an IFN-β luciferase reporter assay in HEK293T cells, we showed that full-length G3BP1 could induce strong IFN-β reporter activity relative to empty vector control (**Figure 4D**). Amongst the truncation mutants examined, G^F^ retained substantial activity, whereas G^E^ showed a weaker effect and the remaining mutants exhibited only limited activity. We then tested these G3BP1 constructs together IRF3 (**Figure 4E**) or TBK1 (**Figure 4F**). When co-expressed with IRF3 or TBK1, G^F^ consistently produced the strongest enhancement of IFN-β reporter activity, whereas G^E^ retained only partial activity and mutants lacking the RGG-motif were largely ineffective.

To assess whether this functional requirement is conserved across species, we repeated the IFN-β reporter assays in RAW264.7 cells. Consistent with the HEK293T cell results, the RGG-containing G3BP1 fragments enhanced IRF3-dependent and TBK1-dependent IFN-β reporter activation, whereas constructs lacking the RGG-containing region were less effective (**Supplementary Figures 5C, D**). These results support the functional relevance of the G3BP1 C-terminal RGG region in both human HEK293T and murine RAW264.7 cells. We also performed parallel IFN-β reporter rescue assays in G3BP1-KO HEK293T cells. Consistent with the results obtained in wild-type cells, G^F_WT^ strongly enhanced IRF3-dependent and TBK1-dependent IFN-β reporter activation, whereas constructs lacking the RGG-containing region showed weaker activity (**Supplementary Figures 6A, B**). These KO-background rescue data support the functional relevance of the C-terminal RGG-containing region in promoting downstream IFN-β signaling. The higher activity of G^F^ compared with G^E^ raises the possibility that the acidic-domain-containing region present in G^E^ but absent in G^F^ may negatively regulate, or conformationally constrain, the RGG-mediated scaffold activity of G3BP1.

These results indicate that the C-terminal RGG domain is the principal motif through which G3BP1 binds TBK1 and IRF3 and supports TBK1/IRF3 association and downstream IFN-β activation.

### 3.5 Arginine methylation of G3BP1 RGG domain promotes its interaction with TBK1

Since RGG domains are known to undergo arginine methylation by protein arginine methyltransferases (PRMTs) (29), we examined if methylation of G3BP1 RGG region contributes to its function during antiviral signaling. Previous studies have implicated arginine methylation of G3BP1 in stress granule biology and Wnt3a–β-catenin signaling (18, 30), raising the possibility that this modification may also regulate its role in innate immune signaling.

**Figure 5:**
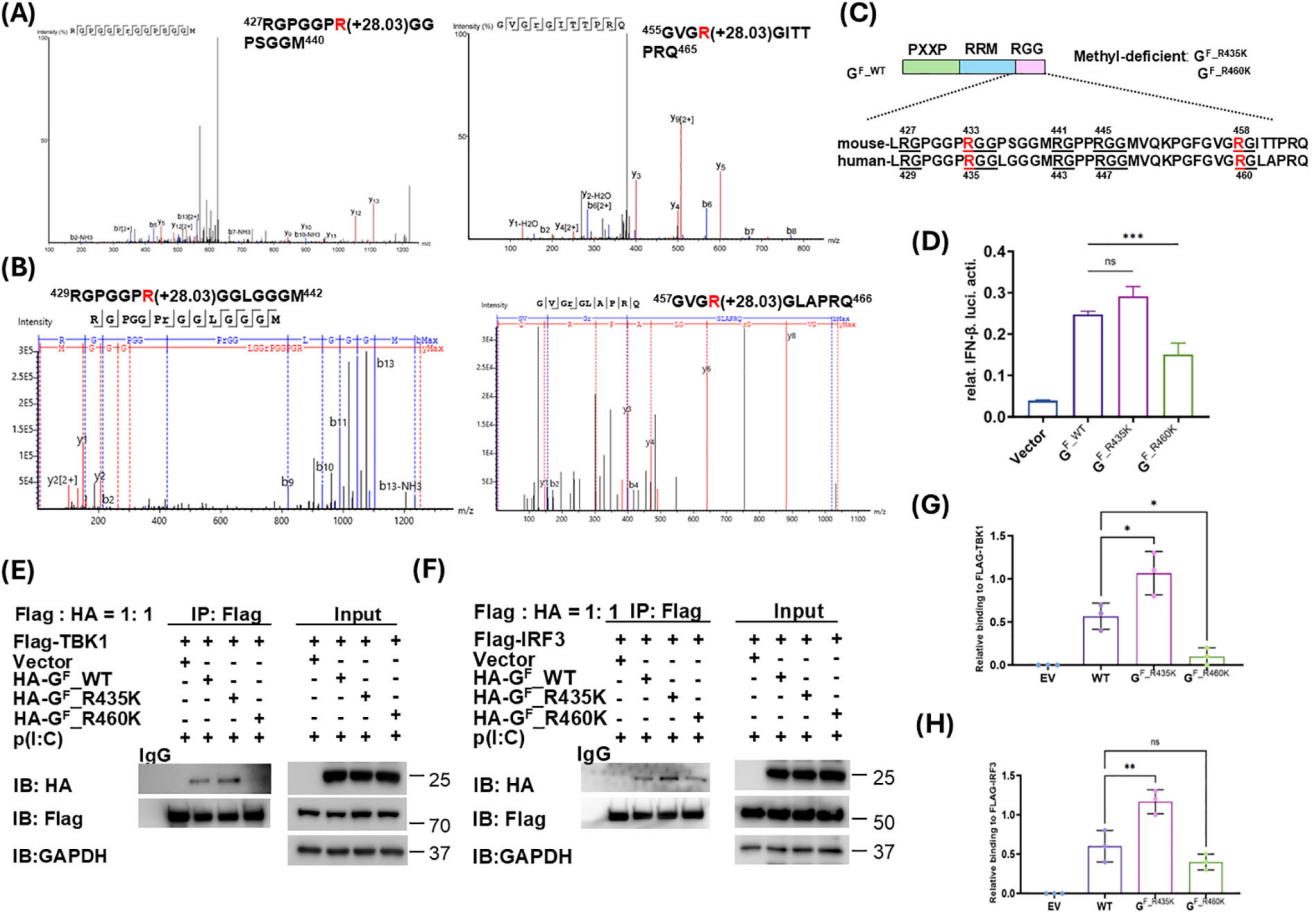
Methylation-associated regulation of the G3BP1 RGG domain promotes TBK1 binding and IFN-β reporter activity. **(A)**, LC–MS/MS analysis of mouse G3BP1 identified dimethylated peptides containing arginine R433 and R458 residues after p(I:C) stimulation of RAW 264.7 cells. **(B)**, LC–MS/MS analysis of FLAG-tagged human G3BP1 over-expressed in HEK293T cells identified p(I:C) dimethylation of arginine R435 and R460 residues within its RGG region. Representative spectra show dimethylated peptides, with a +28.03 Da mass shift indicating dimethylation. **(C)**, Schematic representation of the human G3BP1 G^F^ fragment and alignment of the corresponding mouse and human RGG-region sequences. Conserved arginine residues identified by LC–MS/MS are highlighted, and the human G^F_R435K^ and G^F_R460K^ variants generated for functional analysis are indicated. **(D)**, IFN-β luciferase reporter activity in HEK293T cells transfected with G^F_WT^ or the indicated R435K/R460K variants. Compared with G^F_WT^, the G^F_R460K^ variant showed reduced IFN-β reporter activation. **(E)**, Co-immunoprecipitation analysis of the association between FLAG-TBK1 and HA-tagged G^F_WT^ or the indicated R435K/R460K variants. FLAG-TBK1 was immunoprecipitated, followed by immunoblotting for HA-tagged G3BP1 variants. Input lysates were analyzed as indicated. **(F)**, Co-immunoprecipitation analysis of the association between FLAG-IRF3 and HA-tagged G^F_WT^ or the indicated R435K/R460K variants. FLAG-IRF3 was immunoprecipitated, followed by immunoblotting for HA-tagged G3BP1 variants. Input lysates were analyzed as indicated. Quantification of G3BP1 variant binding to FLAG-TBK1 **(G)** or FLAG-IRF3 **(H)** from independent experiments. Co-immunoprecipitated HA-tagged G3BP1 variants were normalized to immunoprecipitated FLAG-TBK1 or FLAG-IRF3, respectively. Statistical significance was determined as indicated in the graphs. *p ≤ 0.05; **p ≤ 0.01; ***p ≤ 0.001; ns, not significant.

We first performed LC–MS/MS analysis of mouse FLAG-tagged G3BP1 in RAW264.7 cells and identified R433 and R458 within the C-terminal RGG region to be di-methylated (**Figure 5A**). We repeated the LC-MS/MS experiment analyzing FLAG-tagged human G3BP1 in HEK293T cells and found R435 and R460 to be also di-methylated (**Figure 5B**). A schematic alignment of the mouse and human G3BP1 RGG-region sequences illustrates the conservation of these methylated residues, as shown in **Figure 5C**.

To examine the functional importance of these methylation sites, we proceed to generate methylation-deficient arginine-to-lysine (R_K) mutants using the G^F^ construct as the backbone. Specifically, R435K and R460K substitutions were introduced into the human G^F^ fragment, which contains the RGG motif required for interaction with TBK1 and IRF3. We show in an IFN-β luciferase reporter assay that the R460K substitution significantly impaired G3BP1 induction of *ifn-b* activity, whereas the R435K substitution had no effect, when compared with G^F_WT^ (**Figure 5D**). These data indicate that the R460-containing region, and potentially R460 methylation, is functionally important for G3BP1-mediated IFN-β reporter activation. We repeated this reporter analysis in G3BP1-KO HEK293T cells. Consistent with the results obtained in wild-type cells, G^F_R460K^ variant showed impaired IFN-β reporter activation compared with G^F_WT^ and G^F_R435K^ variants (**Supplementary Figure 6C**).

We next assessed whether these methylation-site mutants affect G3BP1 association with TBK1 and IRF3. HEK293T cells were co-transfected with HA-tagged G^F_WT^ or the indicated mutants with Flag–TBK1 or Flag–IRF3, followed by CO-IP. R460K significantly reduced the association of G3BP1 with TBK1 (**Figures 5E, G**). By contrast, R460K did not significantly alter its association with IRF3 (**Figures 5F, H**), indicating that R460 preferentially supports TBK1 engagement rather than the general binding capacity of G3BP1. This selectivity argues against broad disruption of RGG-region binding and suggests that the R460-containing region contributes to the G3BP1–TBK1 interface. Although R435K increased association with both TBK1 and IRF3, this mutation did not impair IFN-β reporter activation. Consistent with the selective defect in TBK1 binding, the reduced IFN-β reporter activity of R460K is most likely attributable to impaired TBK1 recruitment rather than a broad loss of interaction with both signaling partners. Together with the LC–MS identification of dimethylation at the corresponding residue, these findings support an important role for the R460-containing region, and potentially R460 methylation, in promoting efficient recruitment of TBK1 by G3BP1.

As R460K preferentially impaired G3BP1 association with TBK1, we next asked whether this mutation might affect the previously described RIG-I/G3BP1 interaction. We examined the association of RIG-I with HA-tagged G3BP1 RGG domain and the R435K or R460K variants under p(I:C)-stimulated conditions, as shown in **Supplementary Figure 7**. The G3BP1 RGG domain associated with RIG-I, consistent with the reported role of this region in RIG-I binding (6). Notably, neither R435K nor R460K markedly reduced RIG-I/G3BP1 RGG binding. These data suggest that R460 is preferentially required for efficient TBK1 recruitment but is not essential for RIG-I association. Thus, G3BP1-mediated RIG-I engagement and TBK1 recruitment appear to be differentially regulated.

Because R460K impaired G3BP1–TBK1 association, we next examined whether this defect was associated with impaired downstream IRF3 activation in the G3BP1-deficient background. To this end, we evaluated IRF3 nuclear translocation in G3BP1-KO cells reconstituted with full-length G3BP1, G^F_WT^, G^F_R435K^ or G^F_R460K^. Following p(I:C) stimulation, full-length G3BP1, G^F_WT^ and G^F_R435K^ restored IRF3 nuclear accumulation. In contrast, G^F_R460K^ did not efficiently restore IRF3 nuclear translocation. Quantification of nuclear-to-cytoplasmic IRF3 fluorescence intensity ratios confirmed that G^F_R460K^ was defective in restoring IRF3 nuclear translocation compared with full-length G3BP1, G^F_WT^ and G^F_R435K^ (**Supplementary Figure 8**).

Finally, we reintroduced full-length G3BP1, G^F_WT^, G^F_R435K^ or G^F_R460K^ into G3BP1-KO HEK293T cells for functional validations and measured endogenous *IFNB1* mRNA induction after p(I:C) stimulation. Re-expression of full-length G3BP1-WT restored *IFNB1* induction. G^F_WT^ and G^F_R435K^ also increased *IFNB1* expression, whereas G^F_R460K^ failed to restore this response. These results indicate that the R460-containing region is sufficient to restore endogenous *IFNB1* expression in G3BP1-deficient cells (**Supplementary Figure 9**). Together, these mutant and rescue analyses support a model in which the R460-containing region is selectively important for TBK1 recruitment and subsequent IRF3 activation.

### 3.6 The kinase domain of TBK1 binds G3BP1

**Figure 6:**
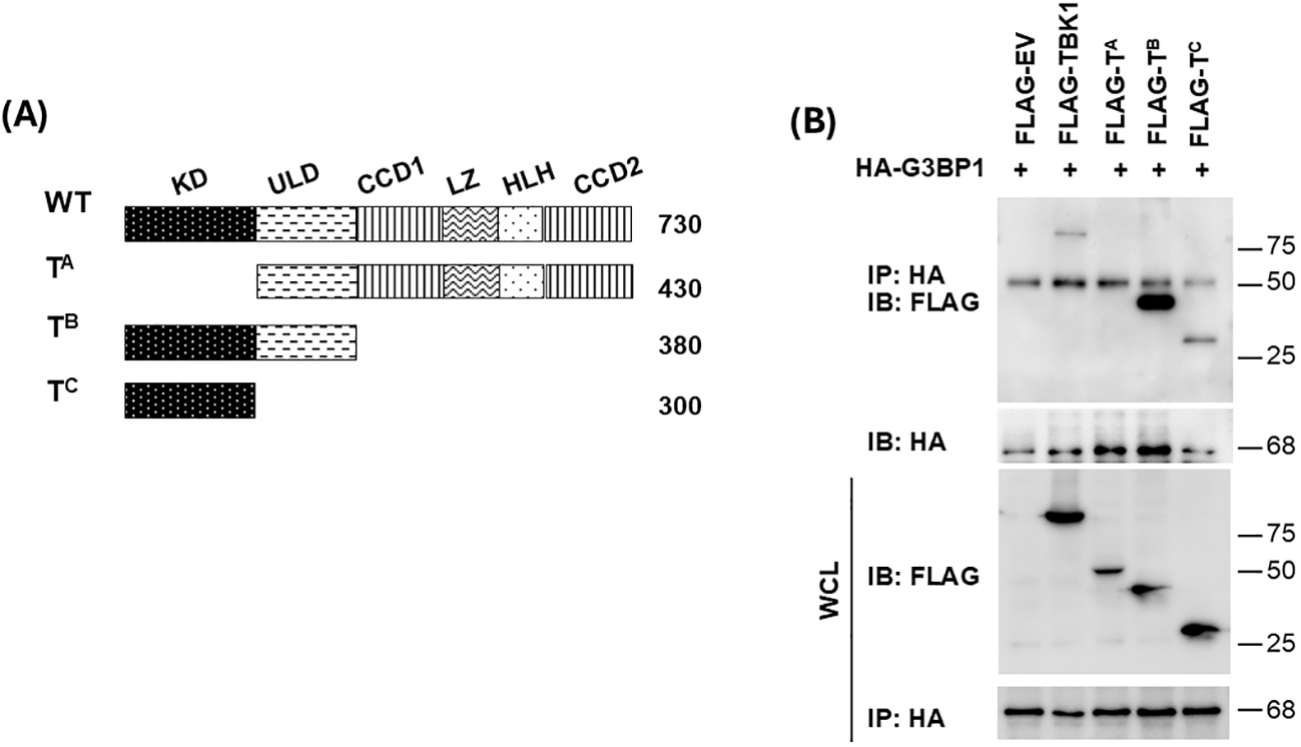
The kinase domain of TBK1 region binds G3BP1. **(A)**, Schematic representation of WT TBK1 and indicated truncation mutants. The amino acid length of each construct is shown on the right. **(B)**, Immunoblot analysis of the interaction between G3BP1 and WT or truncated TBK1 variants. HEK293T cells were co-transfected with HA-tagged G3BP1 together with FLAG-tagged WT or truncation mutants of TBK1. HA–G3BP1 was immunoprecipitated, followed by immunoblotting with the indicated antibodies. Input lysates were analyzed to verify expression of the transfected constructs. A weak, non-specific immunoglobulin heavy-chain signal is visible across the blot.

Having identified methylation of G3BP1 RGG domain, particularly at R460, as important for TBK1 association, we next examined the region of TBK1 that mediates its interaction with G3BP1. To this end, we generated a series of TBK1 truncation mutants spanning distinct structural domains, including constructs lacking the N-terminal kinase domain (KD) or containing the KD with or without the ubiquitin-like domain (ULD) (**Figure 6A**) and over-expressed them in HEK293T cells together with HA-tagged G3BP1. Co-immunoprecipitation analysis showed that full-length TBK1 associated with G3BP1, whereas the T^A^ mutant, which lacks the kinase domain, failed to do so (**Figure 6B**). By contrast, T^B^ and T^C^ variants, both of which retain the kinase domain, could still associate with G3BP1. These findings indicate that TBK1 kinase domain is required, and is likely sufficient, for association with G3BP1.

### 3.7 PRMT5 binds and promotes G3BP1 methylation and its association with TBK1

**Figure 7:**
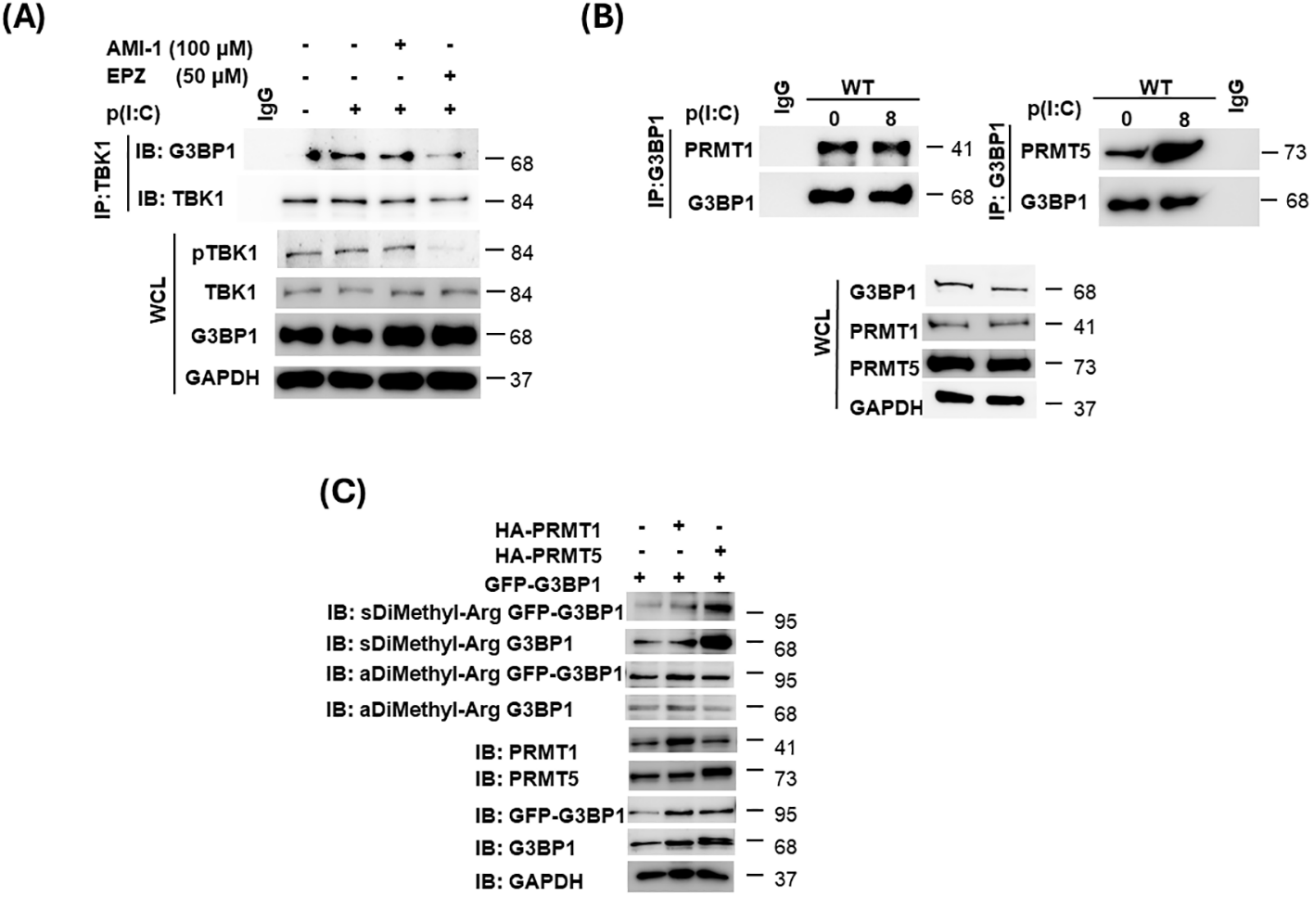
PRMT5 promotes G3BP1 methylation and its association with TBK1. **(A)**, PRMT5 inhibition attenuated the association of G3BP1 with TBK1. HEK293T cells were stimulated with p(I:C) and treated with AMI-1 or EPZ015666 as indicated. TBK1 was immunoprecipitated and immunoblotted for G3BP1. WCL were analyzed for phospho-TBK1, TBK1, G3BP1 and GAPDH. **(B)**, Endogenous interaction of G3BP1 with PRMT1 or PRMT5 in HEK293T cells following p(I:C) stimulation. G3BP1 was immunoprecipitated and immunoblotted for PRMT1 or PRMT5 as indicated. WCL were analyzed for G3BP1, PRMT1, PRMT5 and GAPDH. **(C)**, Cell-based analysis of G3BP1 methylation upon PRMT1 or PRMT5 overexpression. PRMT1, PRMT5 and G3BP1 were overexpressed as indicated, and whole-cell lysates were analyzed by immunoblotting with the corresponding antibodies, including anti-ADMA and anti-SDMA antibodies that recognize asymmetric or symmetric dimethylarginine, respectively. Input lysates were analyzed for GAPDH as indicated.

Protein arginine methyltransferases (PRMTs) catalyze the transfer of methyl groups to arginine residues on target proteins (31). Arginine methylation within the RGG domain emerged as a key determinant of G3BP1–TBK1 association, prompting us to identify the methyltransferase responsible for this regulation. Previous studies have implicated both PRMT1 and PRMT5 in the arginine methylation of G3BP1 (18), raising the possibility that one or both enzymes may contribute to its function in antiviral signaling. To test whether arginine methylation is required for the endogenous association of G3BP1 with TBK1, HEK293T cells were treated with AMI-1 (protein arginine N-methyltransferase inhibitor 1, a pan-PRMT inhibitor) or EPZ015666 (PRMT5 inhibitor) before p(I:C) stimulation. Immunoprecipitation of endogenous TBK1 showed that the specific inhibition of PRMT5 markedly reduced G3BP1 association with TBK1, whereas AMI-1 had little effect under the same conditions (**Figure 7A**). A concomitant decrease in pTBK1 was also observed in the input lysates following EPZ015666 treatment, suggesting that PRMT5 inhibition may impair TBK1 activation in parallel with reducing G3BP1–TBK1 association.

We next examined whether G3BP1 is associated with PRMT1 or PRMT5 during innate immune stimulation. Endogenous G3BP1 was immunoprecipitated from HEK293T cells before or after p(I:C) treatment and immunoblotted for the presence of the two methyltransferases. PRMT1 was detected in G3BP1 immunoprecipitates under both basal and stimulated conditions, with no clear stimulation-dependent increase, whereas association of PRMT5 with G3BP1 was more markedly enhanced after p(I:C) treatment (**Figure 7B**). Incidentally, during our mapping of methylated arginine residues in **Figure 5A**, we had also co-immunoprecipitated PRMT5 with G3BP1 (data not shown). These data suggest that although G3BP1 can associate with both enzymes, PRMT5 is preferentially engaged during antiviral stimulation. Natural infection or p(I:C) stimulation may promote PRMT5-dependent regulation of G3BP1 by enhancing the association or local proximity of PRMT5 and G3BP1, thereby facilitating PRMT5 access to the G3BP1 RGG region.

To assess whether PRMT1 or PRMT5 can promote G3BP1 methylation, we co-expressed GFP–G3BP1 with either PRMT1 or PRMT5 and examined cell lysates using antibodies that recognize either asymmetric dimethylarginine (ADMA) or symmetric dimethylarginine (SDMA). Overexpression of PRMT5 markedly increased the SDMA signal associated with both endogenous G3BP1 and overexpressed GFP–G3BP1, whereas PRMT1 did not enhance ADMA or SDMA signal on either species (**Figure 7C**). This cell-based overexpression assay indicates that PRMT5 is sufficient to promote G3BP1 SDMA modification when expressed at elevated levels. Together with the p(I:C)-induced enhancement of endogenous PRMT5–G3BP1 association, these data suggest that PRMT5-dependent methylation of G3BP1 may be regulated during RNA-triggered signaling through stimulus-dependent recruitment or increased substrate accessibility.

Taken together, these results identify PRMT5 as the methyltransferase most closely linked to G3BP1 methylation during p(I:C)-triggered innate immune signaling. In conjunction with the selective effect of G3BP1 R460K mutant on TBK1 association (**Figure 5**), these findings support a model in which PRMT5-dependent arginine methylation of G3BP1 RGG region promotes the recruitment of TBK1 to G3BP1, thereby facilitating productive coupling of the TBK1–IRF3 signaling axis, leading to IRF3 phosphorylation and activation and the induction of the downstream IFN-β response.

## 4 Discussion

G3BP1 is widely recognized as a core component for the formation of stress granules and has recently been implicated in antiviral functions by binding RIG-I and cGAS (32). Our current study further identifies G3BP1 as a scaffolding protein that promotes productive TBK1–IRF3 coupling, thereby facilitating IRF3 phosphorylation, activation and IFN-β induction. Because IRF3 is regulated by adaptor and scaffold proteins that promote its engagement with upstream kinases such as TBK1 and IKKϵ (33, 34), our findings place G3BP1 within this regulatory layer of IRF3 activation. More broadly, these data support a model in which G3BP1 links RNA recognition, stress adaptation and downstream TBK1–IRF3 signaling.

The TBK1–IRF3 axis is a convergent module downstream of multiple innate immune pathways, including RLR-, TLR3- and cGAS/STING-mediated signaling (35, 36). Although our mechanistic analyses focused on p(I:C)-induced RNA signaling, G3BP1 knockdown reduced *IFNB1* induction in response to both naked and transfected p(I:C), suggesting that G3BP1 acts at a shared step in RNA-sensing pathways. Interestingly, G3BP1 loss impaired IRF3 activation and TBK1–IRF3 association without markedly reducing TBK1 phosphorylation, supporting a role for G3BP1 in coupling activated TBK1 to IRF3 rather than in upstream TBK1 activation.

Complementing this model, time-resolved immunofluorescence clarified the spatial relationship of G3BP1 with IRF3 and TBK1 during p(I:C)-induced signaling. Although IRF3 progressively accumulated in the nucleus after stimulation, G3BP1 remained predominantly cytoplasmic and did not evidently co-translocate with nuclear IRF3 (**Supplementary Figure 2**). In contrast, G3BP1–TBK1 spatial association increased after stimulation, supporting a model in which G3BP1 remains in cytoplasmic signaling compartments to maintain IRF3 association and promote stimulus-induced TBK1 recruitment.

Mechanistically, our results reveal the C-terminal RGG domain of G3BP1 as the principal functional region required for facilitating TBK1/IRF3 association and promoting IFN-β reporter activity in HEK293T cells. These findings were further supported in RAW264.7 cells, where G3BP1 showed stimulus-enhanced TBK1 association and RGG-containing fragments retained IRF3/TBK1 binding and IRF3- or TBK1-dependent IFN-β reporter activity, indicating that the key features of this scaffolding mechanism are conserved across species. The data repeated in the G3BP1-KO background further strengthen the physiological relevance of the domain-mapping experiments.

The domain-mapping experiments also implied that the function of the RGG-containing region may be influenced by adjacent sequences. In particular, the higher IFN-β reporter activity of G^F^ compared with G^E^ suggests that sequences adjacent to the minimal RGG-containing region, including the acidic-domain-containing region, may constrain RGG-mediated scaffolding activity. Although this could reflect autoinhibitory or conformational regulation, alternative effects of truncation on protein stability, folding, localization or motif accessibility cannot be excluded. Targeted acidic-domain deletion and structural analyses will be required to determine whether this region directly represses G3BP1-mediated TBK1–IRF3 signaling.

At the molecular level, the C-terminal RGG region is enriched with arginine-containing RGG/RG repeats, which are canonical substrates for protein arginine methyltransferases (PRMTs) (37). Given that arginine methylation regulates nucleic-acid binding, stress granule dynamics and protein–protein interactions (18, 38), and that RGG domains contribute to stress granule nucleation and structured RNA binding (18, 39), this region may serve as a multifunctional regulatory platform. Consistent with previous studies showing PRMT-dependent regulation of G3BP1 (18, 30), our LC–MS/MS analyses identified methylated residues in both mouse and human G3BP1, including mouse R433/R458 and the corresponding human R435/R460 residues. Functionally, R460K substitution selectively impaired TBK1 association, IRF3 activation and *IFNB1* induction suggesting that R460 is the critical residue for G3BP1-mediated TBK1–IRF3 signaling.

Consistent with the critical role of R460 in IRF3 activation, G3BP1-KO rescue experiments showed that full-length G3BP1, G^F_WT^ and G^F_R435K^, but not G^F_R460K^, restored p(I:C)-induced IRF3 nuclear translocation. The same pattern was observed for downstream functional outputs, as G^F_R460K^ showed impaired restoration of endogenous *IFNB1* induction and reduced IFN-β reporter activation compared with G^F_WT^ or G^F_R435K^ (**Supplementary Figures 6C**, **9**). Although our data suggests that methylation at this site may create or stabilize a selective interface for TBK1 recruitment to phosphorylate IRF3. We should interpret data with caution. Although arginine-to-lysine substitution removes a potential methylation site, it may also alter local conformation, charge distribution, interaction surfaces or introduce lysine-specific modifications such as methylation, acetylation or ubiquitination.

G3BP1 is modulated by both PRMT1 and PRMT5 (18, 40), with PRMT1 reported to methylate R435 and PRMT5 preferentially targeting R460 within the C-terminal RGG region (18, 30). PRMT5 has also been implicated in innate immune regulation in a context- and substrate-dependent manner (41–43). Using AMI-1 and EPZ015666 to inhibit PRMT1 and PRMT5, respectively (44, 45), we found that p(I:C) stimulation enhanced PRMT5–G3BP1 association, whereas PRMT5 inhibition reduced endogenous G3BP1–TBK1 interaction. Consistently, PRMT5 overexpression increased G3BP1 methylation more effectively than PRMT1. These findings support a model in which PRMT5-associated methylation of G3BP1 promotes assembly of a signaling-competent G3BP1–TBK1–IRF3 complex during antiviral stimulation.

This model also helps reconcile the difference between p(I:C)-dependent PRMT5–G3BP1 association and PRMT5-overexpression-induced G3BP1 SDMA. Under endogenous conditions, RNA-triggered signaling may promote G3BP1 methylation by enhancing PRMT5 recruitment, local proximity or RGG-region accessibility. By contrast, forced PRMT5 expression may bypass part of this stimulus-dependent requirement and increased G3BP1 SDMA through elevated enzyme availability. Nevertheless, the precise determinants of PRMT5 substrate specificity toward G3BP1, including possible contributions from cofactors, conformational changes in the RGG region, or additional post-translational modifications, remain important questions for future investigation.

Together with LC–MS identification of R460 methylation, reduced G3BP1–TBK1 interaction after PRMT5 inhibition and PRMT5 mediated increase in G3BP1 SDMA, our data support a model in which the R460 promotes efficient TBK1 recruitment and may be regulated by PRMT5-associated arginine methylation. Future methylation-specific and structural studies will be needed to define how R460 methylation modulates the G3BP1–TBK1 interface.

Our current G3BP1 scaffold model also reconciles present findings with previous reported role of G3BP1 in RIG-I-associated viral RNA sensing. Although RIG-I functions upstream of TBK1–IRF3 signaling, the two events may not represent mutually exclusive or entirely independent processes. Instead, G3BP1 may provide support at multiple levels of the same RNA-triggered antiviral signaling cascade. In this model, G3BP1 may first facilitate RIG-I-associated RNA recognition and then further promote downstream signal propagation by helping to recruit TBK1 to IRF3. Thus, G3BP1 may act as a modular signaling hub that functionally links upstream RNA sensing to downstream IRF3 activation. Together, the time-resolved imaging data provide dynamic support for the model that G3BP1 pre-associates with IRF3 and recruits TBK1 after RNA stimulation, while future time-course biochemical studies will be valuable to further define the kinetics of assembly and disassembly of this signaling complex.

In addition to scaffold-dependent recruitment, TBK1 activity can also be regulated by stress-associated post-translational mechanisms such as oxidation. A recent study reported that TRIM29 promotes PERK SUMOylation and enhances PERK-mediated endoplasmic reticulum stress responses, leading to increased ROS production, TBK1 oxidation and inhibition, thereby suppressing type I IFN production and facilitating viral infection (46). This TRIM29–PERK–ROS–TBK1 oxidation axis provides an important example of how virus-induced cellular stress can negatively regulate TBK1 activity and restrain antiviral IFN signaling. In contrast, our study identifies a methylation-associated scaffold mechanism in which G3BP1 facilitates the recruitment of TBK1 to IRF3. Together, these findings suggest that TBK1–IRF3 signaling is regulated by multiple mechanisms, including oxidative inhibition of TBK1 and methylation-associated assembly of signaling-competent protein complexes.

One limitation of our study is that we focused on G3BP1 and did not directly examine its paralog G3BP2. Given the structural and functional similarity between G3BP1 and G3BP2, future studies using G3BP2-deficient or combined G3BP1/G3BP2 loss-of-function models will be needed to determine whether G3BP2 acts redundantly or cooperatively in RNA-triggered TBK1–IRF3 signaling. It will also be important to test whether the PRMT5–G3BP1–TBK1–IRF3 mechanism extends to other TBK1–IRF3-activating pathways, including those of TLR3 and cGAS/STING.

In summary, our present work uncovers a previously unappreciated role for G3BP1 in promoting antiviral innate immune signaling by facilitating TBK1–IRF3 coupling. Together with the PRMT5 association, SDMA and inhibitor data, these findings support a model in which PRMT5-mediated arginine methylation of G3BP1 RGG domain contributes to efficient TBK1 recruitment and downstream IRF3 activation. These findings broaden the current understanding of G3BP1 as a multifunctional regulator of host defense and suggest that G3BP1 may function as a signalosome to orchestrate anti-viral signaling at multiple steps. Modulation of G3BP1 interactions with RIG-I, TBK1-IRF3 and PRMT5, may represent a potential strategy for tuning antiviral responses.

## Data Availability

The original contributions presented in the study are included in the article/**Supplementary Material**. Further inquiries can be directed to the corresponding author/s.
