# Supplementary material for: G3BP1 methylation by PRMT5 facilitates its recruitment of TBK1 for IRF3 activation during host response to foreign ribonucleic acids
Source: Front Immunol. 2026 Jul 8;17:1888266. doi: 10.3389/fimmu.2026.1888266 (PMC13388048; doi:10.3389/fimmu.2026.1888266)
Supplement: Supplementary file 1 [file Table1.docx]

Supplementary Material

# Supplementary Figures and Tables

## Supplementary Figures


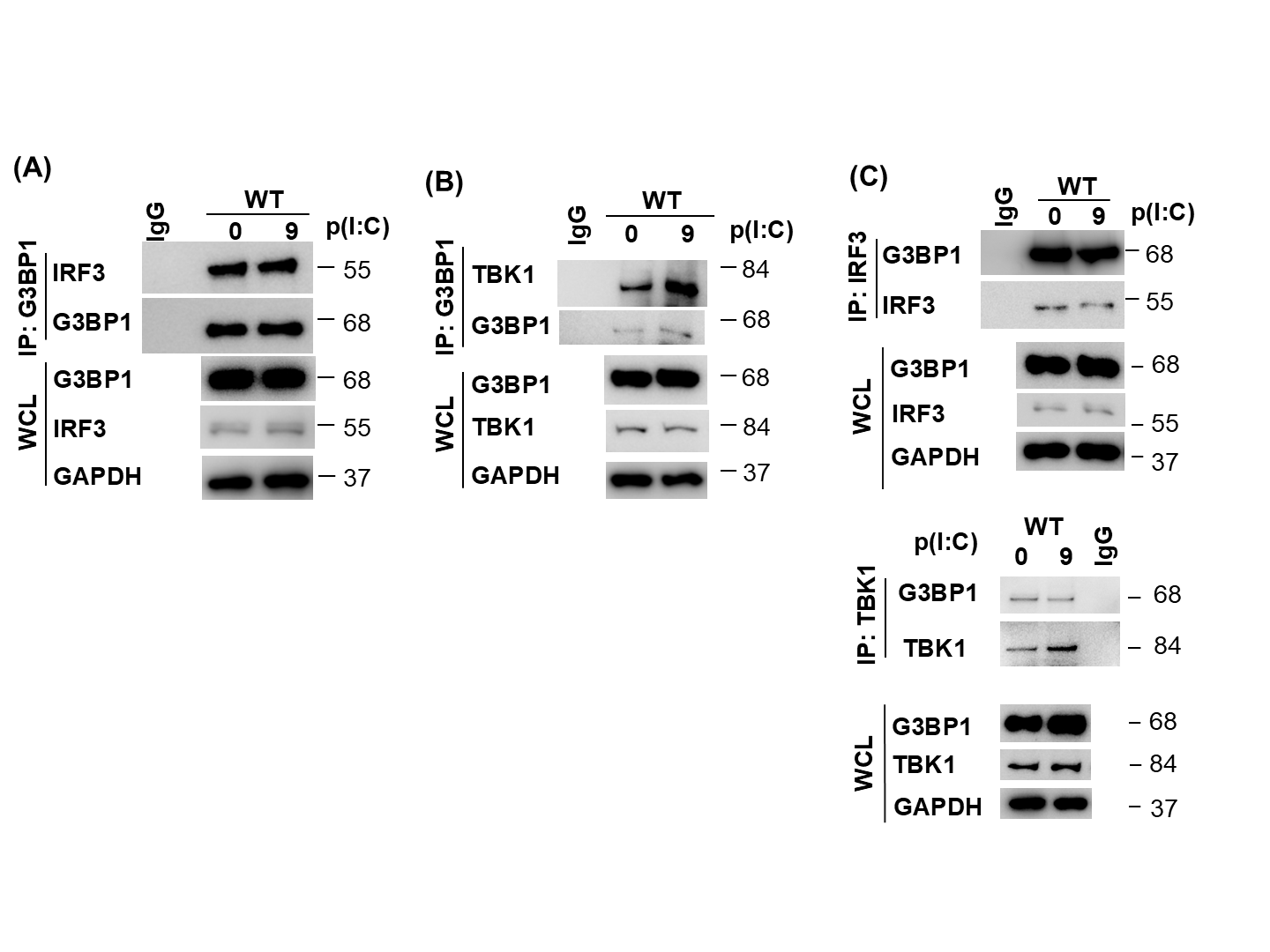


**Supplementary Figure S1 | G3BP1 binds IRF3 constitutively and TBK1 upon p(I:C) stimulation in RAW264.7 cells**. RAW264.7 cells were left untreated or stimulated with p(I:C), and WCL were subjected to Co-IP and immunoblot analysis. **(A)**, G3BP1 immunoprecipitation followed by immunoblotting for IRF3. **(B)**, G3BP1 immunoprecipitation followed by immunoblotting for TBK1. **(C)**, Reciprocal Co-IP using anti-IRF3 or anti-TBK1 antibodies, followed by immunoblotting for G3BP1, IRF3 and TBK1. Input WCL were analyzed for G3BP1, TBK1, IRF3 and GAPDH as indicated.


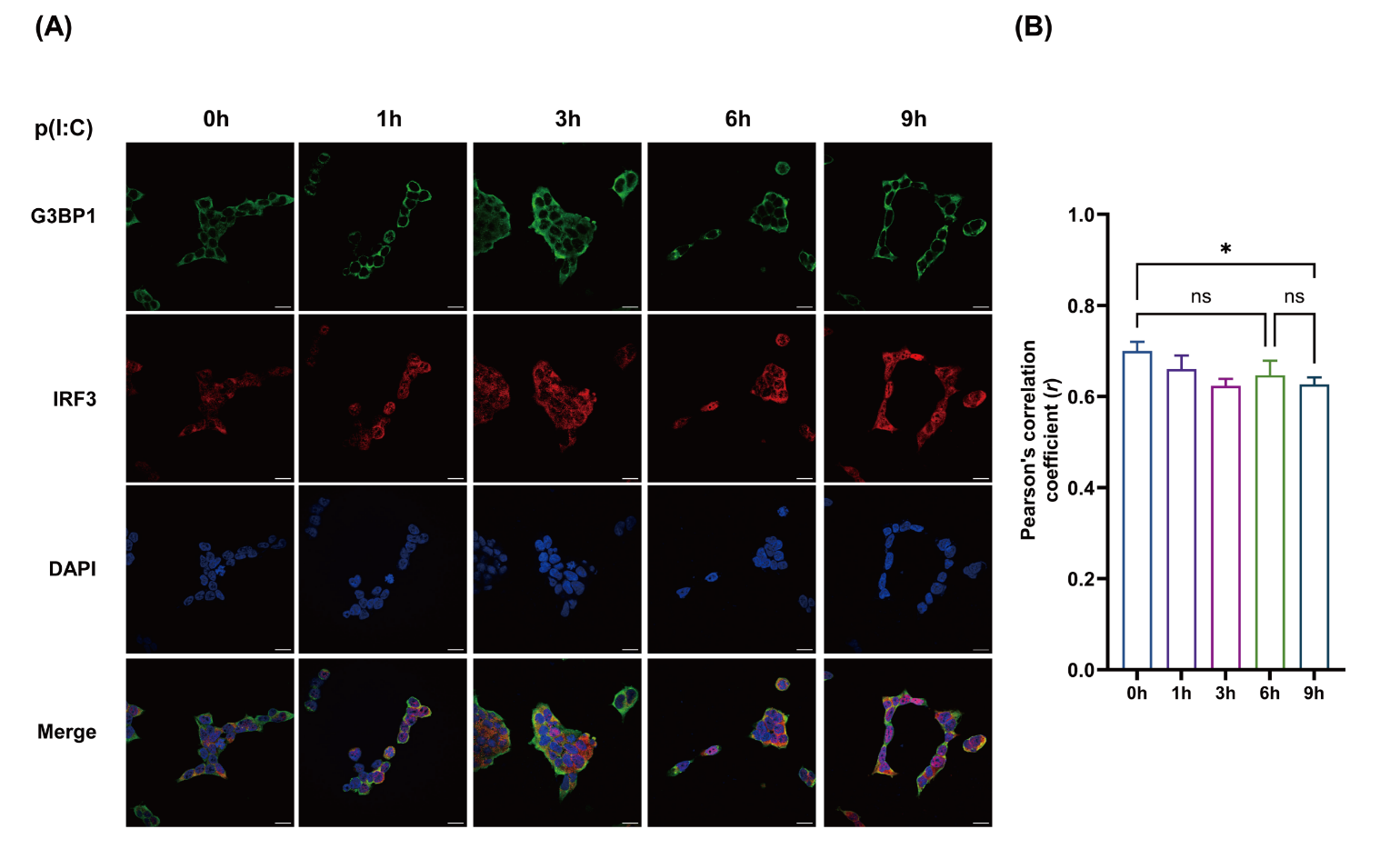


**Supplementary Figure S2 | Time-resolved immunofluorescence analysis of G3BP1 and IRF3 localization following p(I:C) stimulation**. HEK293T cells were left untreated or stimulated with p(I:C) for 0, 1, 3, 6 or 9 h, followed by fixation and immunofluorescence staining for G3BP1 and IRF3. Nuclei were counterstained with DAPI. **(A)**, Representative confocal images showing the subcellular localization and spatial association of G3BP1 and IRF3 at the indicated time points. Scale bars, 20 μm. **(B)**, Quantification of G3BP1–IRF3 spatial association by Pearson’s correlation coefficient at the indicated time points. Data are presented as mean ± SD. Statistical significance was determined as indicated in the graph.


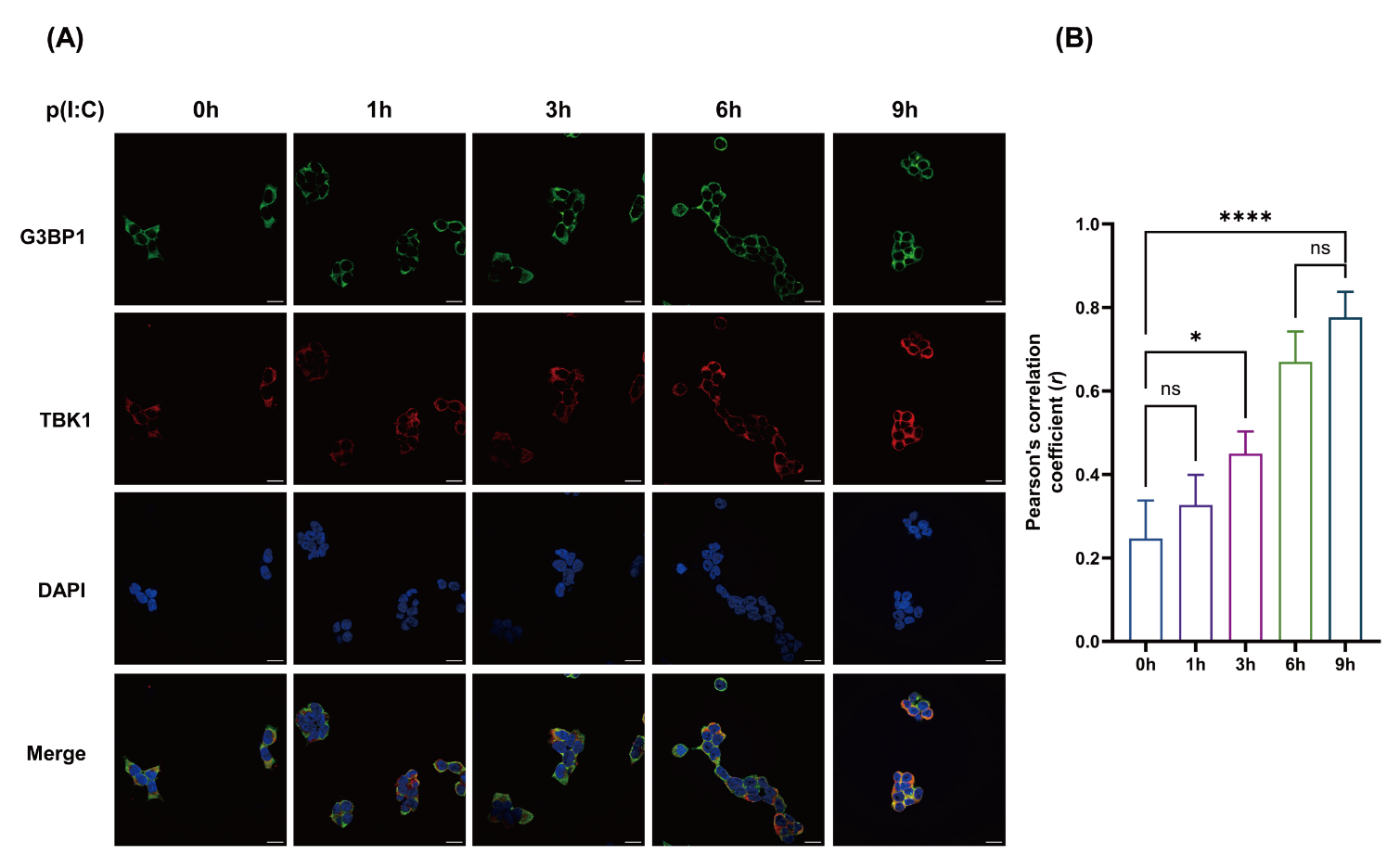


**Supplementary Figure S3 | Time-resolved immunofluorescence analysis of G3BP1 and TBK1 localization following p(I:C) stimulation.** HEK293T cells were left untreated or stimulated with p(I:C) for 0, 1, 3, 6 or 9 h, followed by fixation and immunofluorescence staining for G3BP1 and TBK1. Nuclei were counterstained with DAPI. **(A)**, Representative confocal images showing the subcellular localization and spatial association of G3BP1 and TBK1 at the indicated time points. Scale bars, 20 μm. **(B)**, Quantification of G3BP1–TBK1 spatial association by Pearson’s correlation coefficient at the indicated time points. Data are presented as mean ± SD. Statistical significance was determined as indicated in the graph.


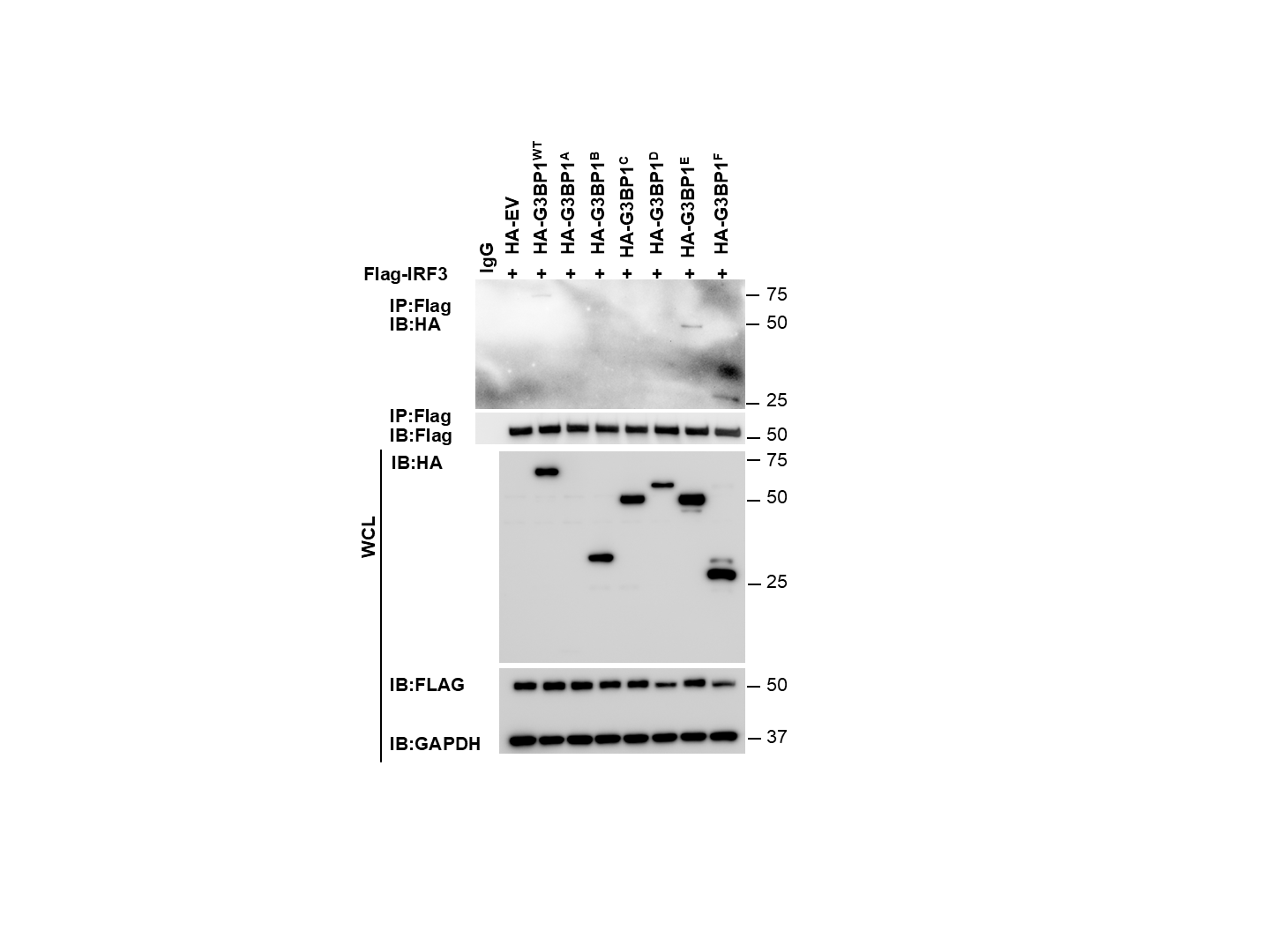


**Supplementary Figure S4 |** **Analysis of IRF3 association with G3BP1 variants in G3BP1-KO HEK293T cells.** G3BP1-KO HEK293T cells were transfected with FLAG-IRF3 together with the indicated HA-tagged G3BP1 truncation variants. FLAG-IRF3 was immunoprecipitated, followed by immunoblotting for HA-tagged G3BP1 variants. Input WCL were analyzed to verify the expression of the transfected constructs.


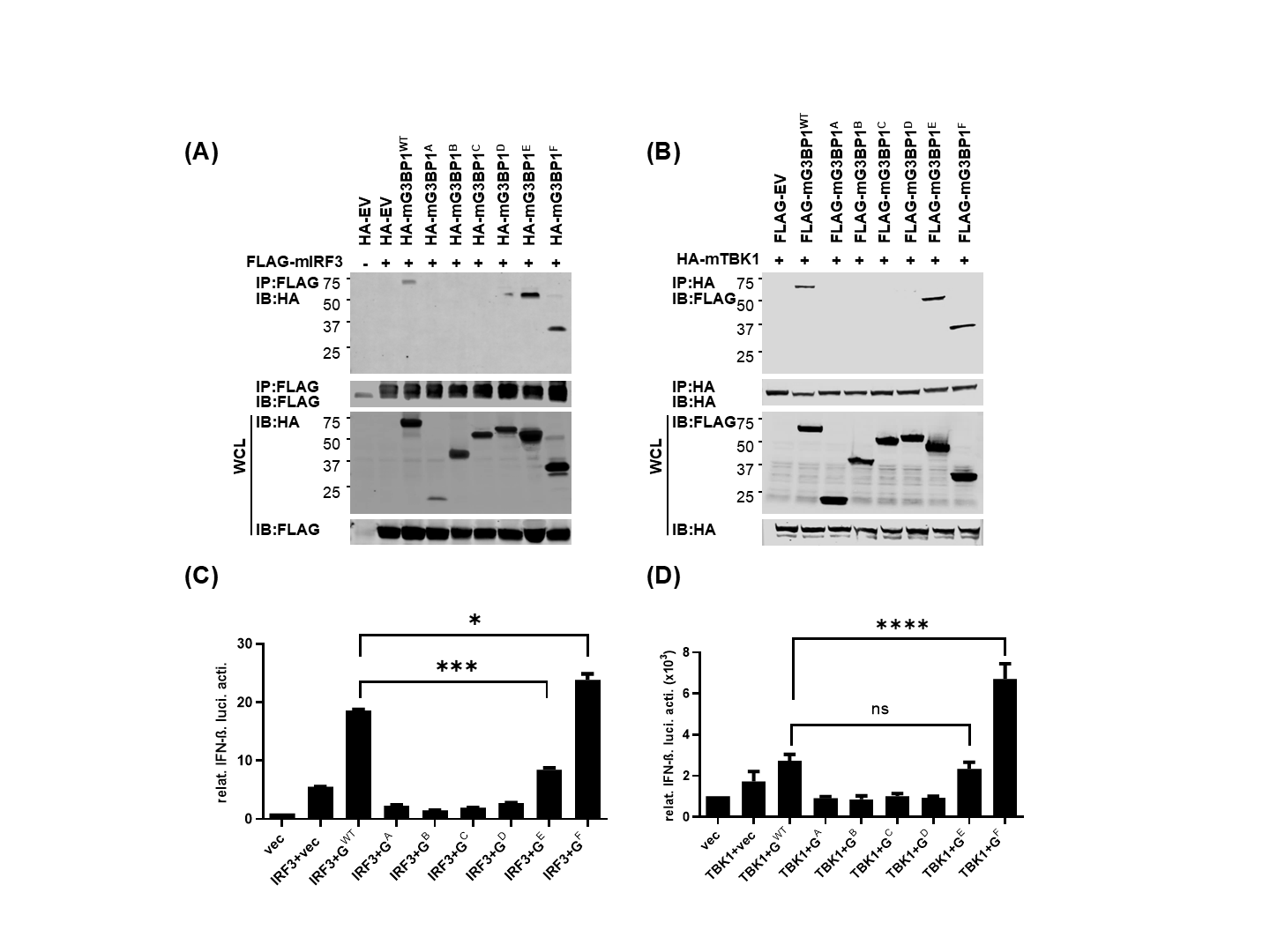


**Supplementary Figure S5 | Validation of G3BP1 variants binding to IRF3/TBK1 and induce of *IFNB1* mRNA production in RAW264.7 cells. (A)**, Co-IP analysis of IRF3 association with mouse G3BP1 truncation variants in RAW264.7 cells. Cells were transfected with FLAG-mIRF3 and the indicated HA-tagged mouse G3BP1 constructs, followed by FLAG immunoprecipitation and immunoblotting with the indicated antibodies. **(B)**, Co-IP analysis of TBK1 association with the indicated mouse G3BP1 truncation variants in RAW264.7 cells. Cells were transfected with HA-mTBK1 and the indicated FLAG-tagged mouse G3BP1 constructs, followed by HA immunoprecipitation and immunoblotting with the indicated antibodies. **(C)**, IFN-β reporter activity in RAW264.7 cells co-transfected with IRF3 and the indicated G3BP1 constructs. **(D)**, IFN-β reporter activity in RAW264.7 cells co-transfected with TBK1 and the indicated G3BP1 constructs. **p* ≤ 0.05; ****p* ≤ 0.001; *****p* ≤ 0.0001; ns, not significant.


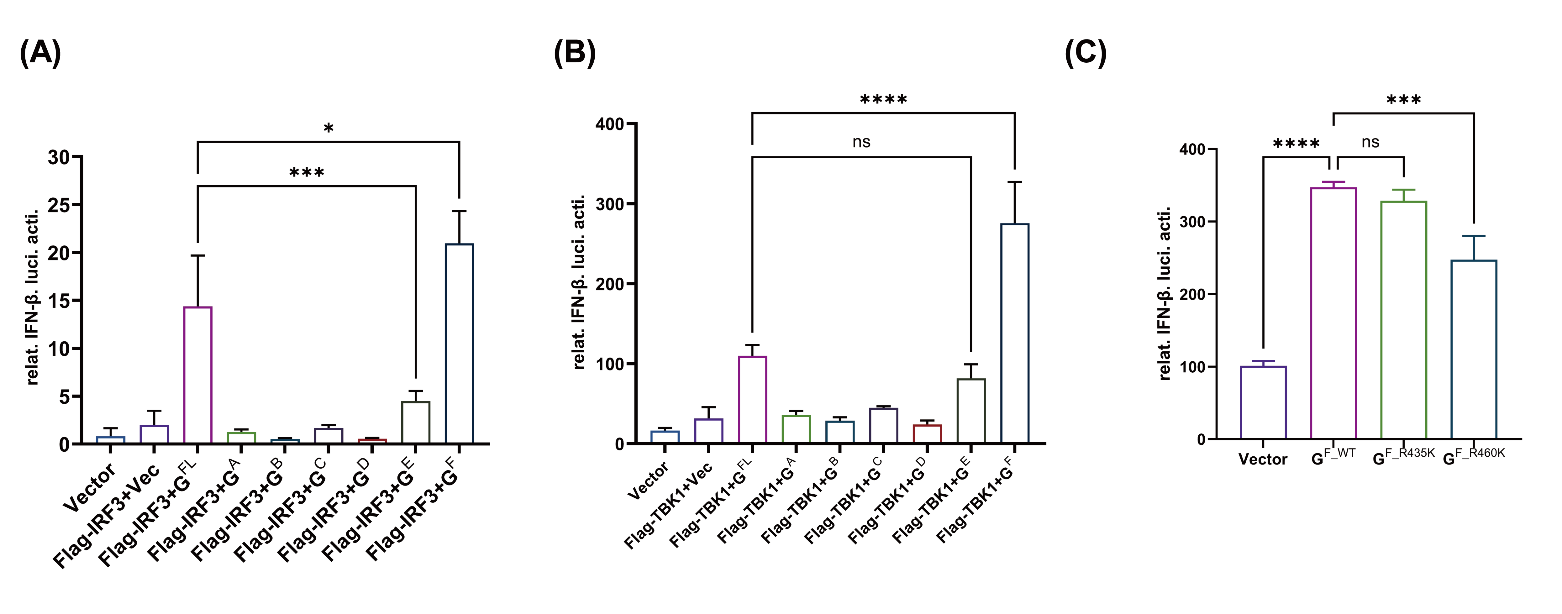


**Supplementary Figure S6 | Functional rescue of IFN-β reporter activity by G3BP1 variants in G3BP1-KO HEK293T cells. (A)**, IFN-β reporter activity in G3BP1-KO HEK293T cells co-transfected with IRF3 and the indicated G3BP1 constructs. **(B)**, IFN-β reporter activity in G3BP1-KO HEK293T cells co-transfected with TBK1 and the indicated G3BP1 constructs. **(C)**, IFN-β reporter activity of G^F_WT^, G^F_R435K^ and G^F_R460K^ in G3BP1-KO HEK293T cells. **p* ≤ 0.05; ****p* ≤ 0.001; *****p* ≤ 0.0001; ns, not significant.


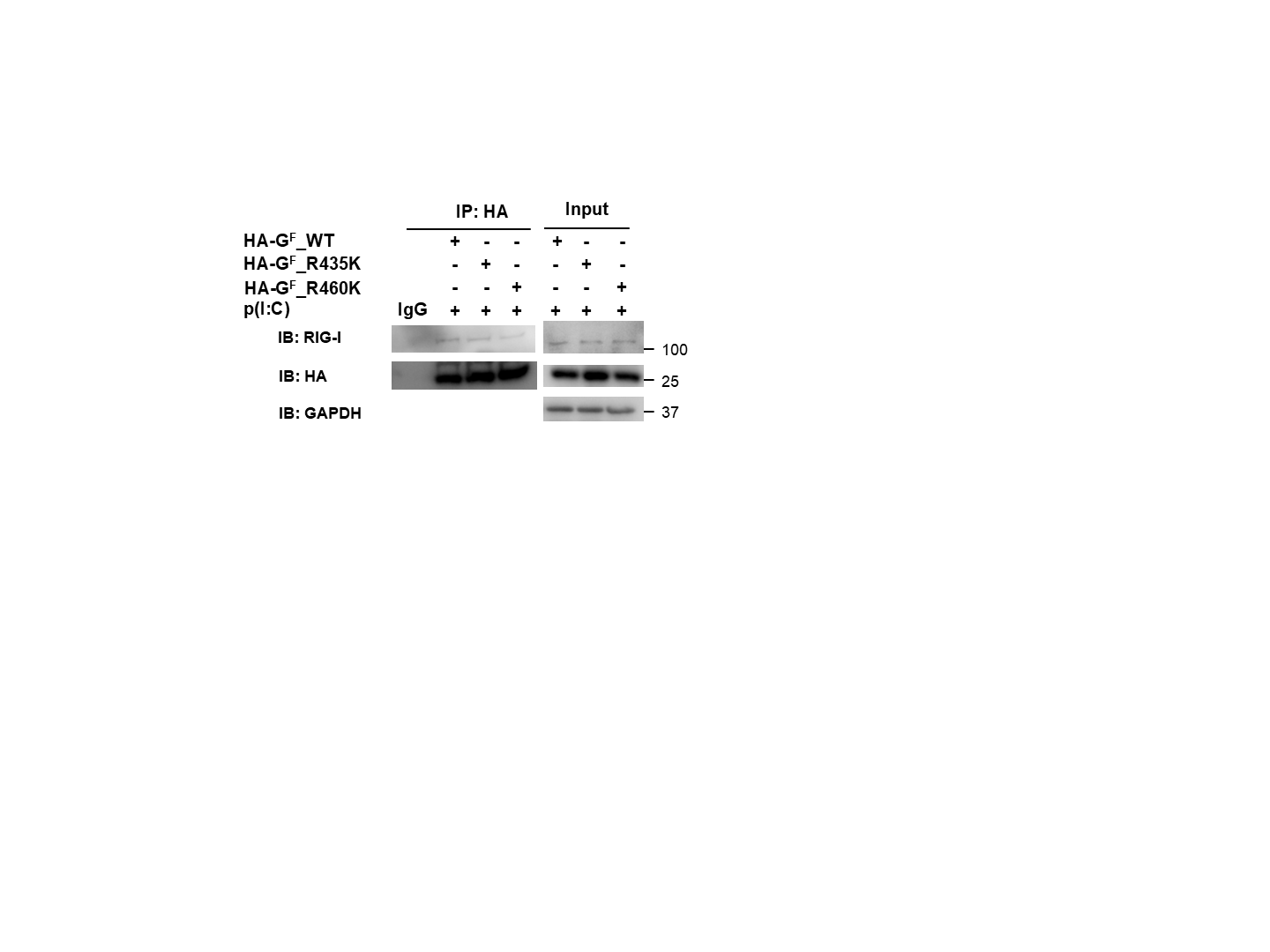


**Supplementary Figure S7 | R460K substitution does not markedly impair the association of the G3BP1 RGG domain with endogenous RIG-I**. HEK293T cells were transfected with HA-tagged G^F_WT^, G^F_R435K^ or G^F_R460K^, followed by p(I:C) stimulation as indicated. WCL were subjected to Co-IP and immunoblot analysis. HA-tagged G3BP1 RGG-domain variants were immunoprecipitated, followed by immunoblotting for endogenous RIG-I. Input WCL were analyzed for HA, RIG-I and GAPDH as indicated. Data shown are representative of independent experiments.


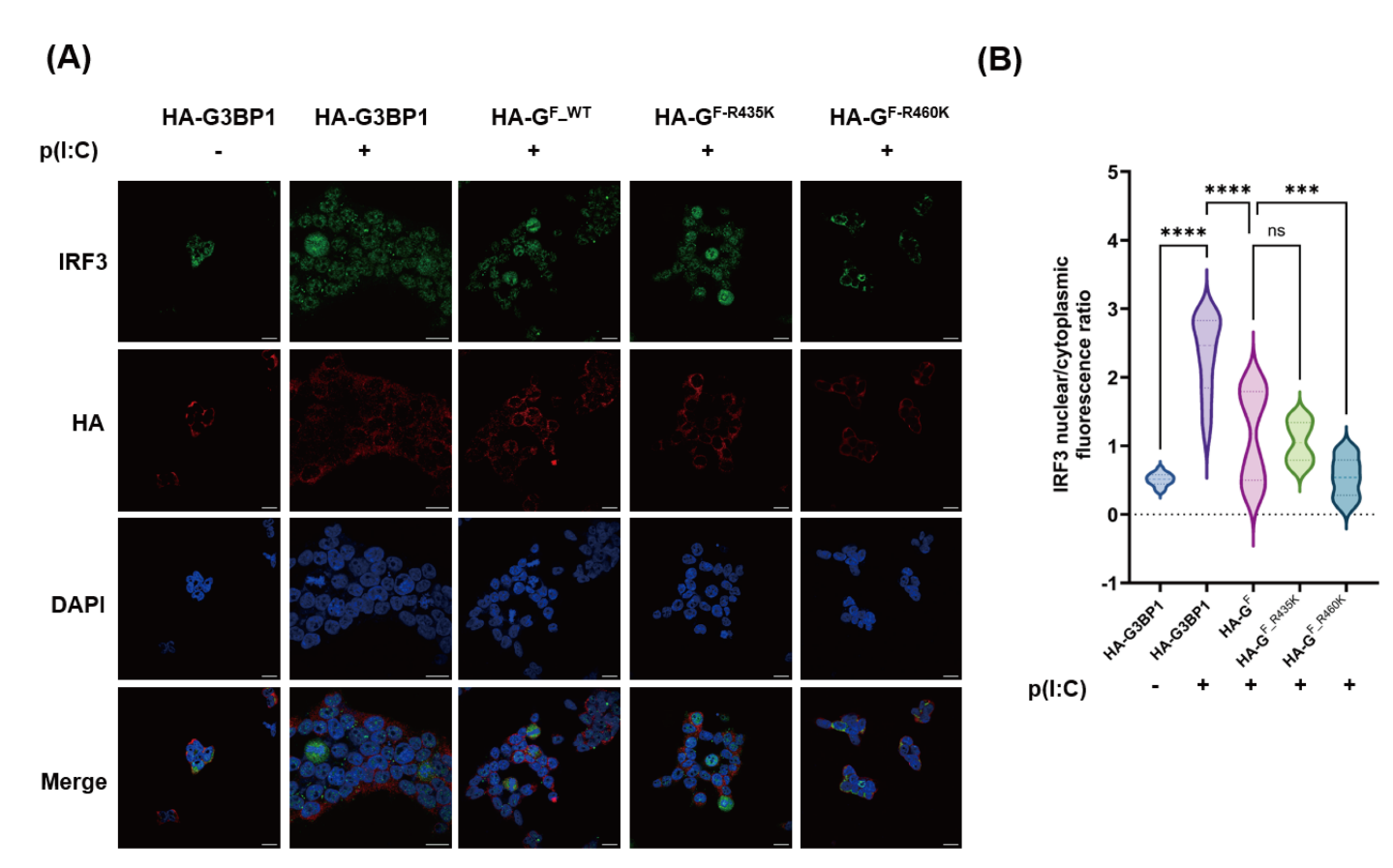


**Supplementary Figure S8 | IRF3 nuclear translocation in G3BP1-KO HEK293T cells transfected with G^F^, G^F_R435K^ and G^F_R460K^ variants.** G3BP1-KO HEK293T cells were reconstituted with HA-tagged full-length G3BP1, HA-G^F_WT^, HA-G^F_R435K^ or HA-G^F_R460K^, followed by p(I:C) stimulation as indicated. Cells were fixed and stained for IRF3 and HA-tagged G3BP1 variants, and nuclei were counterstained with DAPI. **(A)**, Representative confocal images showing IRF3 localization in reconstituted G3BP1-KO cells under unstimulated or p(I:C)-stimulated conditions. HA staining indicates expression of the reintroduced G3BP1 constructs. Scale bars, as indicated. **(B)**, Quantification of nuclear-to-cytoplasmic IRF3 fluorescence intensity ratios under the indicated conditions. Statistical significance was determined as indicated in the graph. ****p* ≤ 0.001; *****p* ≤ 0.0001; ns, not significant.


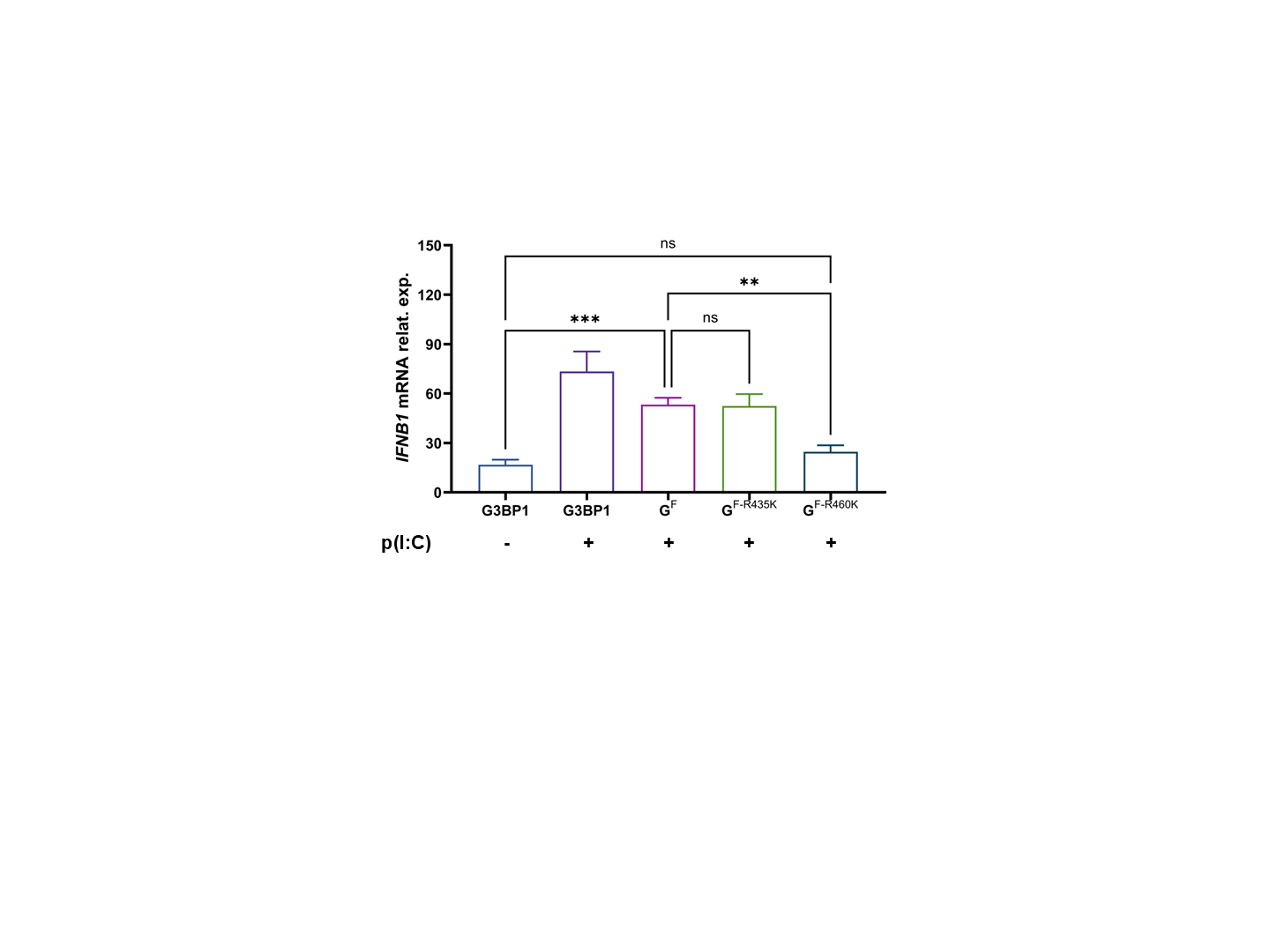


**Supplementary Figure S9 | Rescue of endogenous *IFNB1* mRNA induction by G3BP1 variants in G3BP1-KO HEK293T cells.** G3BP1-KO HEK293T cells were reconstituted with full-length G3BP1, G^F_WT^, G^F_R435K^ or G^F_R460K^, followed by p(I:C) stimulation as indicated. Endogenous *IFNB1* mRNA expression was measured by qRT-PCR. ***p* ≤ 0.01; ****p* ≤ 0.001; ns, not significant.

## Supplementary Tables

**Supplementary Table 1**. Primer sequences used for amplification and cloning of G3BP1, TBK1 and IRF3 expression constructs, generation of G3BP1 (G^A^–G^F^) and TBK1 (T^A^–T^C^) truncation mutants, and site-directed mutagenesis of G3BP1 GF_^R435K^ and GF_^R460K^ constructs.

| Vector |  | Forward primer | Reverse primer |
| --- | --- | --- | --- |
| pXJ40-HA (Cat. No.: V013136) | G3BP1-Full length | acgcaggatccaagcttATGGTGATGGAGAAGCCT | ataagatctggtaccTTACTGCCGTGGCGCAA |
|  | G3BP1-A | cgcaggatccaagcttATGGTGATGGAGAAGC | aagatctggtaccTTAAAACCCACCAAAG |
|  | G3BP1-B | ccggatccaagcttATGGTGATGGAGAAGC | taagatctggtaccTTACTCAGGGGCAGTTTCTT |
|  | G3BP1-C | tccggatccaagcttATGGTGATGGAGAAG | aataagatctggtaccTTAACTGTCAGGGTG |
|  | G3BP1-D | tccggatccaagcttATGGTGATGGAGAAGCCT | ttaataagatctggtaccTTAATTCAGACGGACCT |
|  | G3BP1-E | gatccaagcttGTCACTGAGCCTCAGG | ataagatctggtaccTTACTGCCGTGGCGC |
|  | G3BP1-F | ccggatccaagcttGATGCTCAGAAGAGTTCTT | aagatctggtaccTTACTGCCGTGGCG |
|  | G3BP1-F-R435K | GAGGCCCTAAAGGTGGGCTGGGTG | AGCCCACCTTTAGGGCCTCCAGGT |
|  | G3BP1-F-R460K | AGTGGGAAAAGGGCTTGCGCCACGGC | AGTGGGAAAAGGGCTTGCGCCACGGC |
| Pxj40-Flag (Cat. No.: V013093) | IRF3 | gatccaagcttATGGGAACCCCAAAGC | aagatctggtaccTCAGCTCTCCCCA |
|  | TBK1 | tccggatccaagcttATGCAGAGCACTTCT | ataagatctggtaccCTAAAGACAGTCAAC |
|  | TBK1-A | tccggatccaagcttATTCATGTTTTTTC | agatctggtaccCTAAAGACAGTCAACGT |
|  | TBK1-B | cggatccaagcttATGCAGAGCACTT | gatctggtaccTTATTCCCGGCTTACTA |
|  | TBK1-C | tccggatccaagcttATGCAGAGCACTTC | agatctggtaccTTATACCATTCGGTGAA |
| For qRT-PCR | *IFNB1* | TTCAGTGTCAGAAGCTCCTGTGG | CTGCTTAATCTCCTCAGGGATGTCA |
| For qRT-PCR | *GAPDH* | GAAGGTGAAGGTCGGAGTC | GAAGATGGTGATGGGATTTC |
